# Presynaptic cAMP-PKA-mediated potentiation induces reconfiguration of synaptic vesicle pools and channel-vesicle coupling at hippocampal mossy fiber boutons

**DOI:** 10.1371/journal.pbio.3002879

**Published:** 2024-11-18

**Authors:** Olena Kim, Yuji Okamoto, Walter A. Kaufmann, Nils Brose, Ryuichi Shigemoto, Peter Jonas

**Affiliations:** 1 Institute of Science and Technology Austria (ISTA), Klosterneuburg, Austria; 2 Department of Molecular Neurobiology, Max Planck Institute for Multidisciplinary Sciences, Göttingen, Germany; Universität Regensburg, GERMANY

## Abstract

It is widely believed that information storage in neuronal circuits involves nanoscopic structural changes at synapses, resulting in the formation of synaptic engrams. However, direct evidence for this hypothesis is lacking. To test this conjecture, we combined chemical potentiation, functional analysis by paired pre-postsynaptic recordings, and structural analysis by electron microscopy (EM) and freeze-fracture replica labeling (FRL) at the rodent hippocampal mossy fiber synapse, a key synapse in the trisynaptic circuit of the hippocampus. Biophysical analysis of synaptic transmission revealed that forskolin-induced chemical potentiation increased the readily releasable vesicle pool size and vesicular release probability by 146% and 49%, respectively. Structural analysis of mossy fiber synapses by EM and FRL demonstrated an increase in the number of vesicles close to the plasma membrane and the number of clusters of the priming protein Munc13-1, indicating an increase in the number of both docked and primed vesicles. Furthermore, FRL analysis revealed a significant reduction of the distance between Munc13-1 and Ca_V_2.1 Ca^2+^ channels, suggesting reconfiguration of the channel-vesicle coupling nanotopography. Our results indicate that presynaptic plasticity is associated with structural reorganization of active zones. We propose that changes in potential nanoscopic organization at synaptic vesicle release sites may be correlates of learning and memory at a plastic central synapse.

## Abstract 2

Information storage in neuronal circuits is hypothesized to involve nanoscopic structural changes at synapses, but direct evidence for this is lacking. This study uses nanophysiology and functional electron microscopy to reveal structural changes of hippocampal active zones during chemical potentiation.

## Introduction

Synapses are key sites of exchange and storage of information in the brain [1]. Transmitter release has been extensively characterized by biophysical techniques, going back to classical work at the neuromuscular junction [2] and more recent studies at the calyx of Held in the auditory brainstem [3]. In parallel, synaptic structure has been characterized in much detail by light and electron microscopy (EM) [4,5]. However, the connection between synaptic biophysics and morphology remains unclear. For example, biophysical analysis often determines the number of functional release sites, but the structural correlates have not been identified [6]. Furthermore, biophysical measurements report the coupling distance between Ca^2+^ channels and release sensors [7,8] but the physical correlate of this measure remains undefined. Finally, biophysical studies often delineate different vesicle pools at presynaptic active zones (AZs), specialized sites of synaptic vesicles fusion and neurotransmitter release [9–13] but how these pools correlate to vesicle populations in electron micrographs or tomograms remains unclear. For example, the readily releasable pool (RRP) often closely correlates with the pool of docked vesicles (i.e., vesicles in the direct contact with AZ plasma membrane) [14] but exceptions to this correlation have been also observed [15–17]. Further correlated biophysical-structural approaches are needed to address these questions.

Transmitter release at central synapses is not constant but undergoes substantial activity-dependent changes [18]. Structural changes associated with synaptic plasticity are of particular interest, because they may represent parts of “engrams,” defined as physical, chemical, or structural changes underlying information storage in the brain [19]. However, because of the nanoscopic scale of the modifications, the nature of these engrams has long remained elusive [20]. Recent work suggested that presynaptic short-term potentiation associated with an increase in RRP and release probability (P_r_, the probability of synaptic vesicle (SV) fusion with the plasma membrane) leads to the formation of “pool engrams” [21]. However, changes in Ca^2+^ channel localization or channel–vesicle coupling may occur in parallel and could affect the P_r_ of SVs. Moreover, whether similar changes in RRP occur during more long-lasting forms of plasticity or through the effects of neuromodulators remains to be determined [22]. To understand the precise nature of synaptic engrams, a nanoscale analysis of the topographical arrangement of presynaptic Ca^2+^ channels and docked SVs before and after plasticity induction is needed. Although recent work demonstrated the feasibility of such a technically demanding approach [23], rigorous correlated biophysical and morphological analysis of defined glutamatergic synapses at the single-synapse level to resolve the nanoscale changes during presynaptic plasticity induction is lacking.

The hippocampal mossy fiber–CA3 pyramidal neuron (PN) synapse, formed between dentate gyrus granule cells (GCs) and CA3 PNs, is an ideal synapse to tackle these questions [24,25]. First, it is suitable for direct presynaptic–postsynaptic recording [26], which enables precise biophysical analysis of transmission at the single-synapse level. Second, it has a unique extent of presynaptic plasticity, including facilitation, post-tetanic potentiation (PTP) [21], and long-term potentiation (LTP) [27]. Third, the plasticity at hippocampal mossy fiber synapses is dependent on well-defined canonical signaling pathways. For example, high-frequency stimulation (HFS) induces several cyclic adenosine monophosphate (cAMP)-dependent forms of presynaptic plasticity [21,28–33]. Similarly, forskolin, an adenylyl cyclase (AC) activator, leads to marked chemical potentiation [29,32,33]. Finally, it is accessible to quantitative structural analysis by EM [34,35], high-pressure freezing (HPF) [36–38], and freeze-fracture replica labeling (FRL) [39].

Using combined nanophysiological and ultrastructural analysis, we found that AC activation by forskolin increased the size of the RRP and the docked vesicle pool in parallel. Furthermore, we discovered that forskolin slightly reduced the distance between Ca_V_2.1 (P/Q-type) Ca^2+^ channels and Munc13-1, an essential vesicle priming protein and putative marker of primed vesicles [14,40–45]. Taken together, our results provide novel insights into the mechanisms of chemical potentiation at central synapses and the nanoscopic mechanisms of engram formation.

## Results

### Activation of the cAMP-PKA signaling pathway increases RRP and P_r_

HFS-induced potentiation has been demonstrated to increase the RRP size [21]. However, the mechanisms of chemical potentiation remain controversial. Recent work suggested an increase in number of release sites [46] or an accumulation of presynaptic Ca^2+^ channels [47] but delineating the exact mechanisms requires precise analysis of synaptic transmission at the unitary level.

**Fig 1:**
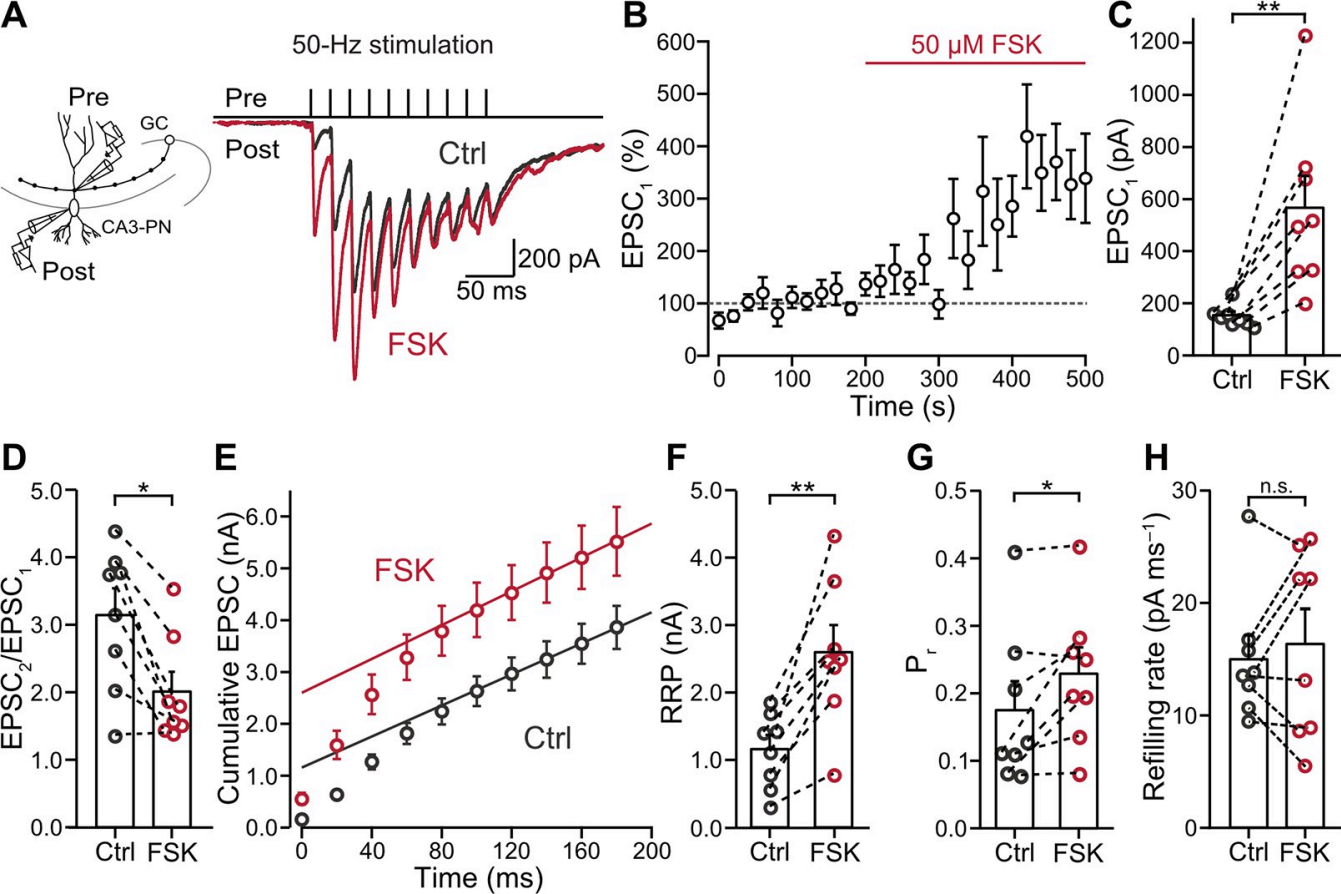
Forskolin-induced chemical potentiation is primarily mediated by an increase in RRP size. ** (A)** Left panel: schematic illustration of the paired recording. Single MFBs were stimulated in the tight-seal, cell-attached configuration, while postsynaptic CA3-PNs were simultaneously recorded in the whole-cell voltage-clamp configuration. Right top panel: 50-Hz train of 10 stimuli. Right bottom panel: overlay of average EPSCs before (“Ctrl,” gray) and in the presence of 50 μM forskolin (“FSK,” red). **(B)** Normalized EPSC_1_ peak amplitude plotted against experimental time. Red horizontal line indicates the application of forskolin (“FSK”). Dashed line indicates 100% baseline. Note that the onset of forskolin application is the time point of switching solutions from forskolin-free ACSF to forskolin-containing ACSF. Forskolin-containing ACSF reached the recording chamber about 60–90 s after the onset of forskolin application. Data from 8 pairs (8 rats). **(C)** Summary bar graph of EPSC_1_ peak amplitudes before (“Ctrl,” gray) and in the presence of 50 μM forskolin (“FSK,” red). Bars and whiskers show mean + SEM; *P* = 0.0078, Wilcoxon signed-rank test. **(D)** Summary bar graph of PPR (EPSC_2_/EPSC_1_) before (“Ctrl,” gray) and in the presence of 50 μM forskolin (“FSK,” red). Bars and whiskers show mean + SEM; *P* = 0.0156, Wilcoxon signed-rank test. **(E)** Cumulative plot of EPSC peak amplitudes during a 50-Hz train with 10 stimuli before (“Ctrl,” gray) and in the presence of 50 μM forskolin (“FSK,” red). Data points during the last 4 stimuli (at time points ≥120 ms) were fit by linear regression and back-extrapolated to time point 0. **(F–H)** Summary bar graphs of RRP (F; *P* = 0.0078), P_r_ (G; *P* = 0.0391), and refilling rate (H; *P* = 0.7422, Wilcoxon signed-rank tests), estimated from the cumulative EPSC plot (E), before (“Ctrl,” gray) and in the presence of 50 μM forskolin (“FSK,” red). Bars and whiskers show mean + SEM. See S1 and S2 Figs. Numerical values for this figure are detailed at https://doi.org/10.15479/AT:ISTA:18296. ACSF, artificial cerebrospinal fluid; EPSC, excitatory postsynaptic current; MFB, mossy fiber bouton; PN, pyramidal neuron; PPR, paired-pulse ratio; RRP, readily releasable pool; SEM, standard error of the mean.

To pinpoint the biophysical mechanisms of chemical potentiation at hippocampal mossy fiber synapses, we performed paired recordings from presynaptic mossy fiber boutons (MFBs) and postsynaptic CA3 PNs (Figs 1 and S1). To maximally activate the cAMP pathway, we applied forskolin, a widely used AC activator [28–31,33,48]. A 5-min application of 50 μM forskolin caused a potentiation of evoked excitatory postsynaptic currents (EPSCs) at MFB–CA3 PN synapses to 368% of control amplitude (Fig 1A–1C; EPSC_1_ –control: 154.1 ± 15.1 pA (mean ± SEM), median 146.8 pA; forskolin: 566.9 ± 122.3 pA, median 511.8 pA, *n* = 8 pairs and *N* = 8 rats in all analyses; control versus forskolin: *P* = 0.0078, Wilcoxon signed-rank test). These results confirm that chemical potentiation by forskolin markedly increases the strength of mossy fiber synaptic transmission at the single-synapse level.

To determine the locus of chemical potentiation, we analyzed the paired-pulse ratio (PPR; EPSC_2_/EPSC_1_; Fig 1D). PPR significantly decreased after forskolin application (EPSC_2_/EPSC_1_ –control: 3.14 ± 0.40 (mean ± SEM), median 3.47; forskolin: 2.01 ± 0.29, median 1.70; control versus forskolin: *P* = 0.0156, Wilcoxon signed-rank test), consistent with a presynaptic locus of potentiation and an increase in P_r_. Furthermore, we analyzed amplitude and frequency of miniature EPSCs (mEPSCs; S2 Fig). mEPSCs frequency but not amplitude was affected by forskolin (S2D–S2G Fig, mEPSCs frequency–control: 3.09 ± 0.62 Hz (mean ± SEM), median 2.07 Hz; forskolin: 4.33 ± 0.54 Hz, median 4.05 Hz; control versus forskolin: *P* = 0.0185; mEPSCs amplitude control: 24.7 ± 1.5 pA, median 23.6 pA; forskolin: 23.6 ± 1.0 pA, median 23.1 pA; control versus forskolin: *P* = 0.4131, Wilcoxon signed-rank test; *n* = 11 cells and *N* = 3 rats), indicating no change in postsynaptic glutamate sensitivity. These results suggest a presynaptic locus of forskolin-induced potentiation.

To dissect the biophysical mechanisms of forskolin potentiation, we performed analysis of cumulative EPSC amplitudes (Fig 1E) [11,21,49]. This method can distinguish between changes in RRP and P_r_, although the resulting RRP values represent “pool decrement” rather than absolute pool size ([11]; see Materials and methods). Notably, we found that both RRP and P_r_ significantly increased after forskolin application (Fig 1F and 1G; RRP–control: 1.16 ± 0.21 nA (mean ± SEM), median 1.27 nA; forskolin: 2.60 ± 0.40 nA, median 2.50 nA; control versus forskolin: *P* = 0.0078, Wilcoxon signed-rank test; P_r_ control: 0.17 ± 0.04 (mean ± SEM), median 0.12; forskolin: 0.23 ± 0.04, median 0.22; control versus forskolin: *P* = 0.0391, Wilcoxon signed-rank test). In contrast, the refilling rate remained unchanged after forskolin application (Fig 1H; refilling rate–control: 15.0 ± 2.1 pA ms^−1^ (mean ± SEM), median 13.6 pA ms^−1^; forskolin: 16.3 ± 3.1 pA ms^−1^, median 17.5 pA ms^−1^; control versus forskolin: *P* = 0.7422, Wilcoxon signed-rank test). Thus, chemical potentiation primarily involved an increase in pool size but also an increase in P_r_, reminiscent of hippocampal mossy fiber PTP [21].

**Fig 2:**
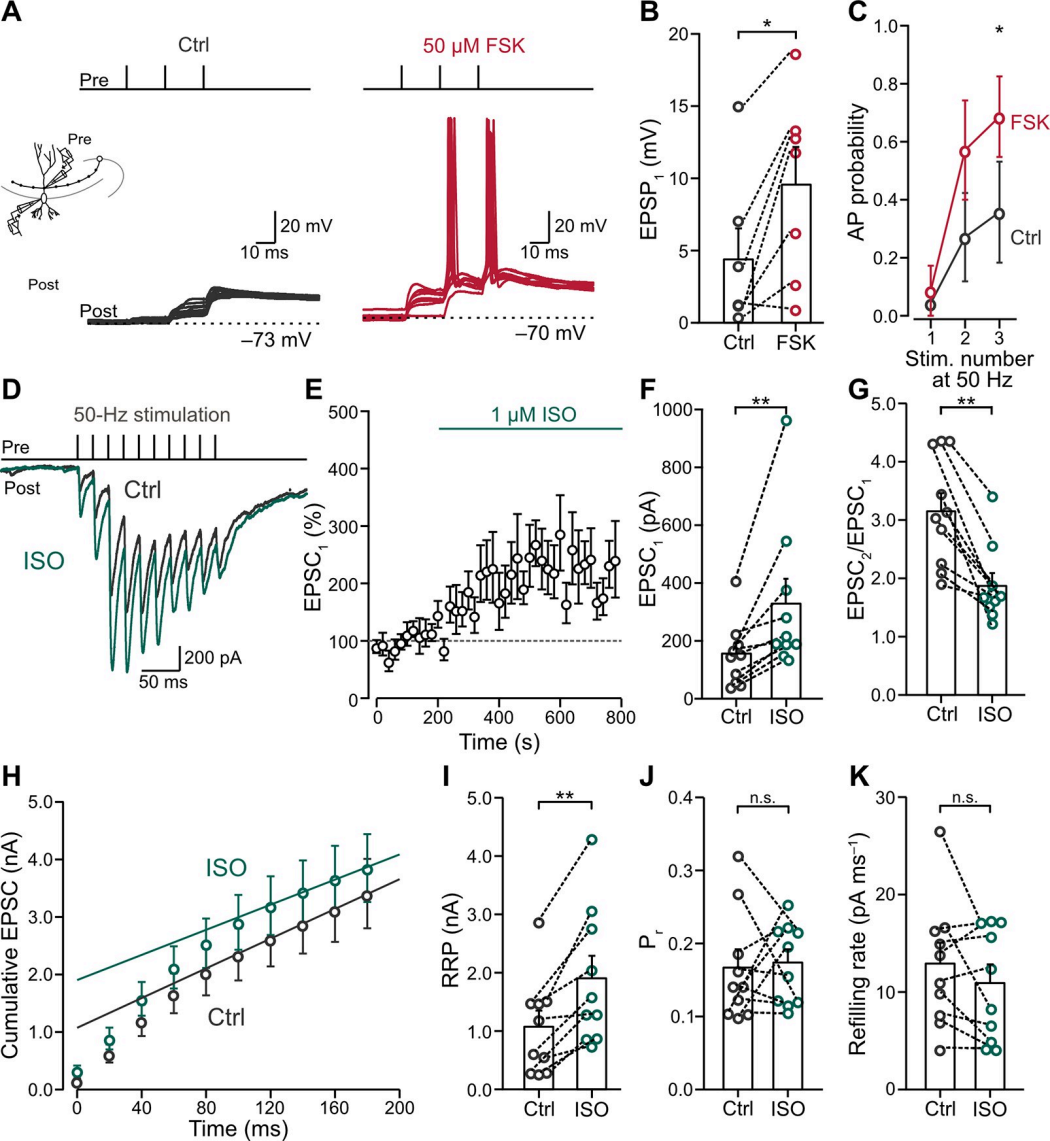
The effects of natural cAMP-PKA activation in MFBs. **(A)** Left inset: schematic illustration of the paired recording. Single MFBs were stimulated in the tight-seal, cell-attached configuration, while postsynaptic CA3-PNs were simultaneously recorded in the whole-cell current-clamp configuration. Top panels: 50-Hz train of 3 stimuli. Bottom panels: representative traces of EPSPs and APs in CA3-PNs before (gray) and after 50 μM forskolin (red). **(B)** Summary bar graph of EPSP_1_ peak amplitude before (“Ctrl,” gray) and in the presence of 50 μM forskolin (“FSK,” red). Bars and whiskers show mean + SEM; *P* = 0.0313, Wilcoxon signed-rank test. **(C)** Probability of AP firing before (“Ctrl,” gray) and in the presence of 50 μM forskolin (“FSK,” red). EPSP_3_: *P* = 0.0313, Wilcoxon signed-rank test. **(D)** Top panel: 50-Hz train of 10 stimuli. Bottom panel: representative average EPSCs before (“Ctrl,” gray) and in the presence of 1 μM isoproterenol (“ISO,” green). Recording configuration similar to Fig 1A. **(E)** Normalized EPSC_1_ peak amplitude plotted against experimental time. Green horizontal line indicates the application of isoproterenol (“ISO”). Dashed line indicates 100% baseline. The switch of the solution is similar to Fig 1B. Data from 10 pairs in 8 rats. **(F)** Summary bar graph of EPSC_1_ peak amplitudes before (“Ctrl,” gray) and in the presence of 1 μM isoproterenol (“ISO,” green). Bars and whiskers show mean + SEM; *P* = 0.0020, Wilcoxon signed-rank test. **(G)** Summary bar graph of PPR (EPSC_2_/EPSC_1_) before (“Ctrl,” gray) and in the presence of 1 μM isoproterenol (“ISO,” green). Bars and whiskers show mean + SEM; *P* = 0.0020, Wilcoxon signed-rank test. **(H)** Cumulative plot of EPSC peak amplitudes during a 50-Hz train with 10 stimuli before (“Ctrl,” gray) and in the presence of 1 μM isoproterenol (“ISO,” green). Data points during the last 4 stimuli (at time points ≥120 ms) were fit by linear regression and back-extrapolated to time point 0. **(I–K)** Summary bar graphs of readily RRP (I; *P* = 0.0020), P_r_ (J; *P* = 0.8457), and refilling rate (K; *P* = 0.3750, Wilcoxon signed-rank tests), estimated from the cumulative EPSC plot (H), before (“Ctrl,” gray) and in the presence of 1 μM isoproterenol (“ISO,” green). Bars and whiskers show mean + SEM. Numerical values for this figure are detailed at https://doi.org/10.15479/AT:ISTA:18296. AP, action potential; cAMP, cyclic adenosine monophosphate; EPSC, excitatory postsynaptic current; EPSP, excitatory postsynaptic potential; MFB, mossy fiber bouton; PN, pyramidal neuron; PPR, paired-pulse ratio; RRP, readily releasable pool; SEM, standard error of the mean.

To further assess the functional significance of chemical potentiation, we tested the effects of forskolin on unitary excitatory postsynaptic potentials (EPSPs; Fig 2A–2C). Forskolin increased the amplitude of the first EPSP in a 50-Hz train (EPSP_1_; Fig 2B; EPSP_1_ –control: 4.39 ± 1.99 mV (mean ± SEM), median 1.35 mV; forskolin: 9.56 ± 2.42 mV, median 11.89 mV, *n* = 7 pairs and *N* = 7 rats; control versus forskolin: *P* = 0.0313, Wilcoxon signed-rank test). However, the amplitude of EPSP_1_ remained below the threshold for action potential (AP) initiation in both conditions. In contrast, for the second and third EPSP (EPSP_2_ and EPSP_3_) in a 50-Hz train, the AP probability significantly increased after forskolin application (Fig 2C; probability of third AP–control: 0.36 ± 0.17 (mean ± SEM); forskolin: 0.69 ± 0.14, *n* = 7 pairs and *N* = 7 rats; control versus forskolin: *P* = 0.0313, Wilcoxon signed-rank test). Thus, chemical potentiation markedly regulates the conditional detonation properties of the synapse [25,50,51].

To test whether a more natural activation of the cAMP-PKA signaling pathway had similar effects, we applied 1 μM isoproterenol, a β_1_ and β_2_ adrenergic receptor agonist, known to activate AC via G_s_ proteins (Fig 2D–2K) [52–54]. Similar to forskolin, isoproterenol led to potentiation of EPSC_1_ in 10 out of 13 synapses (Fig 2D–2F; EPSC_1_ –control: 155.9 ± 36.7 pA (mean ± SEM), median 154.2 pA; isoproterenol: 329.0 ± 85.7 pA, median 209.3 pA, *n* = 10 pairs and *N* = 8 rats; control versus isoproterenol: *P* = 0.0020, Wilcoxon signed-rank test), while the rest remained unpotentiated (control: 261.3 ± 28.1 pA (mean ± SEM), median 252.3 pA; isoproterenol: 166.9 ± 18.3 pA, median 176.2 pA, *n* = 3 pairs and *N* = 3 rats; control versus isoproterenol: *P* = 0.1235, paired *t* test). To analyze the underlying mechanisms of isoproterenol-induced potentiation, we focused on the group of potentiated synapses. To further test for the presynaptic locus of chemical potentiation induced by isoproterenol, we analyzed the PPR (Fig 2G). PPR significantly decreased after isoproterenol application (EPSC_2_/EPSC_1_ –control: 3.15 ± 0.31 (mean ± SEM), median 3.06; isoproterenol: 1.87 ± 0.22, median 1.68; control versus isoproterenol: *P* = 0.0020, Wilcoxon signed-rank test), again consistent with a presynaptic locus of potentiation. Further cumulative EPSC analysis revealed that the potentiation led to an increase in RRP size (Fig 2H and 2I; RRP–control: 1.08 ± 0.28 nA (mean ± SEM), median 0.92 nA; isoproterenol: 1.90 ± 0.39 nA, median 1.46 nA; control versus isoproterenol: *P* = 0.0020, Wilcoxon signed-rank test). However, neither P_r_ nor refilling rate changed after induction of chemical potentiation with isoproterenol (Fig 2J and 2K; P_r_−control: 0.17 ± 0.02 (mean ± SEM), median 0.14; isoproterenol: 0.17 ± 0.02, median 0.18; control versus isoproterenol: *P* = 0.8457, Wilcoxon signed-rank test; refilling rate–control: 12.9 ± 2.1 pA ms^−1^ (mean ± SEM), median 12.5 pA ms^−1^; isoproterenol: 10.9 ± 1.9 pA ms^−1^, median 10.5 pA ms^−1^; control versus isoproterenol: *P* = 0.3750, Wilcoxon signed-rank test). These results indicate that natural activation of the cAMP-PKA signaling pathway leads to expansion of the RRP, although with subtle differences and larger variability than direct AC activation by forskolin.

### Chemical potentiation leads to an expansion of the docked vesicle pool

**Fig 3:**
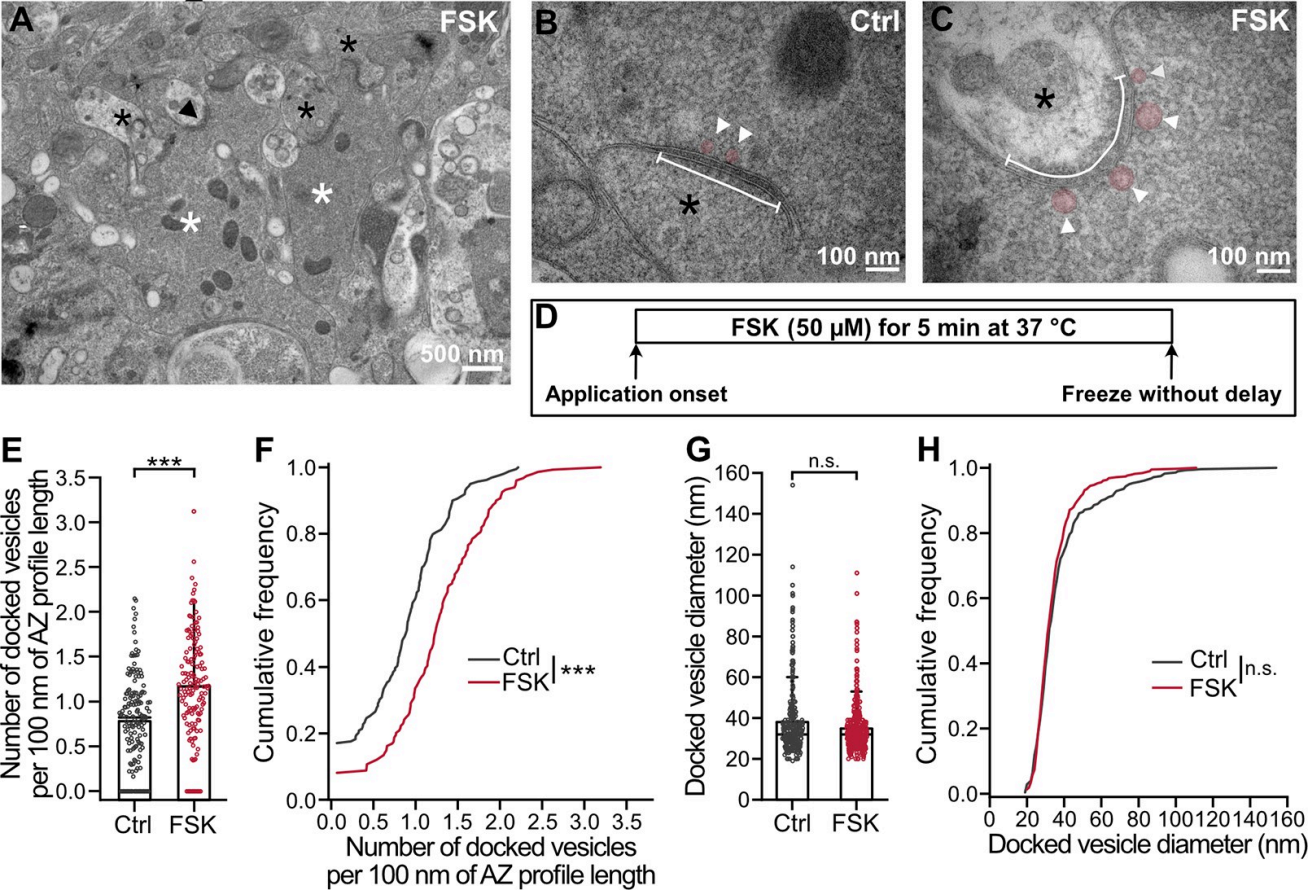
Docked vesicle pool of MFBs expands after induction of chemical potentiation. **(A)** Example TEM micrograph from acute hippocampal slices, showing putative MFBs (white asterisks) with apparent postsynaptic spines (black asterisks) in *stratum lucidum* of CA3 region. MFBs were recognized based on characteristic morphological features: large bouton size, high density of clear SVs, presence of large dense-core vesicles, high-density of mitochondria, and multiple synaptic contacts with large spines. Black arrowhead points to an AZ shown in (C). **(B, C)** Higher magnification TEM micrographs showing AZs (white line) and docked SVs (pink circles and white arrowheads) in putative MFBs in DMSO control (B, “Ctrl”) and after 50 μM forskolin (C, “FSK”). **(D)** Schematic representation of the time course of the experiment with 5-min forskolin (“FSK”) treatment. **(E)** Summary bar graph of the number of docked vesicles per 100 nm of AZ profile length in DMSO control (“Ctrl,” gray) and after forskolin application (“FSK,” red). Note zero values indicate AZs without any observed docked vesicles. Bars and whiskers show mean + SD. Horizontal black lines indicate median values. *P* < 0.0001, Mann–Whitney test. **(F)** Cumulative plots of the data displayed in (E), color scheme is identical to (E). *P* < 0.0001, Mann–Whitney test. **(G)** Summary bar graph of the diameter of docked vesicles measured in DMSO control (“Ctrl,” gray) and after forskolin treatment (“FSK,” red). Bars and whiskers show mean + SD. Horizontal black lines indicate median values. *P* = 0.1689, Mann–Whitney test. **(H)** Cumulative plots of the data displayed in (G), color scheme is identical to (G). *P* = 0.2317, Mann–Whitney test. Scale bar sizes are indicated on the figure panels. Numerical values for this figure are detailed at https://doi.org/10.15479/AT:ISTA:18296. AZ, active zone; DMSO, dimethyl sulfoxide; MFB, mossy fiber bouton; SD, standard deviation; SV, synaptic vesicle; TEM, transmission electron microscopy.

Considering the robust effects of forskolin on RRP and P_r_, we next wanted to test whether any structural changes occur in the AZ at the vesicle docking level at MFB–CA3 PN synapses (Fig 3). We therefore cryo-fixed acute hippocampal slices with and without forskolin treatment (50 μM; 5 min) and performed freeze-substitution (Fig 3A–3D). Forskolin was used in this set of experiments, because of the reliability of the effects and the previously documented compatibility with EM experiments [22,46,55]. Vesicles whose outer membrane was in direct contact with the presynaptic AZ membrane were considered “docked.” After forskolin application, the number of docked SVs increased in comparison to control conditions (Fig 3E; number of docked vesicles per 100 nm AZ profile length–control: 0.78 ± 0.52 (mean ± SD), median 0.82, *n* = 159 AZ, *N* = 3 mice; forskolin: 1.20 ± 0.60, median 1.17, *n* = 149 AZ, *N* = 3 mice; control versus forskolin: *P* < 0.0001, Mann–Whitney test). This change was also evident in cumulative distributions, where the forskolin sample group was shifted towards the right (Fig 3F; control versus forskolin: *P* < 0.0001, Mann–Whitney test). In contrast, the diameter of docked vesicles did not change upon application of forskolin (Fig 3G and 3H; docked vesicle diameter–control: 38.1 ± 17.9 nm (mean ± SD), median 32.0 nm, *n* = 159 AZ, *n* = 280 vesicles, *N* = 3 mice; forskolin: 34.9 ± 12.2 nm, median 32.0 nm, *n* = 149 AZ, *n* = 382 vesicles, *N* = 3 mice; control versus forskolin: *P* = 0.1689, Mann–Whitney test; control versus forskolin: *P* = 0.2317, Mann–Whitney test). The effects of forskolin on the docked vesicle pool were comparable to those observed during HFS-induced mossy fiber PTP [21]. However, the effects on vesicle diameter were distinct, because docked vesicle diameter was constant in the case of forskolin potentiation but slightly increased in the case of HFS-induced PTP [21]. Thus, direct activation of the cAMP-PKA signaling pathway affected the docking of smaller and larger SVs to the same extent.

### Chemical potentiation affects the topographical relation between Ca_V_2.1 and Munc13-1

**Fig 4:**
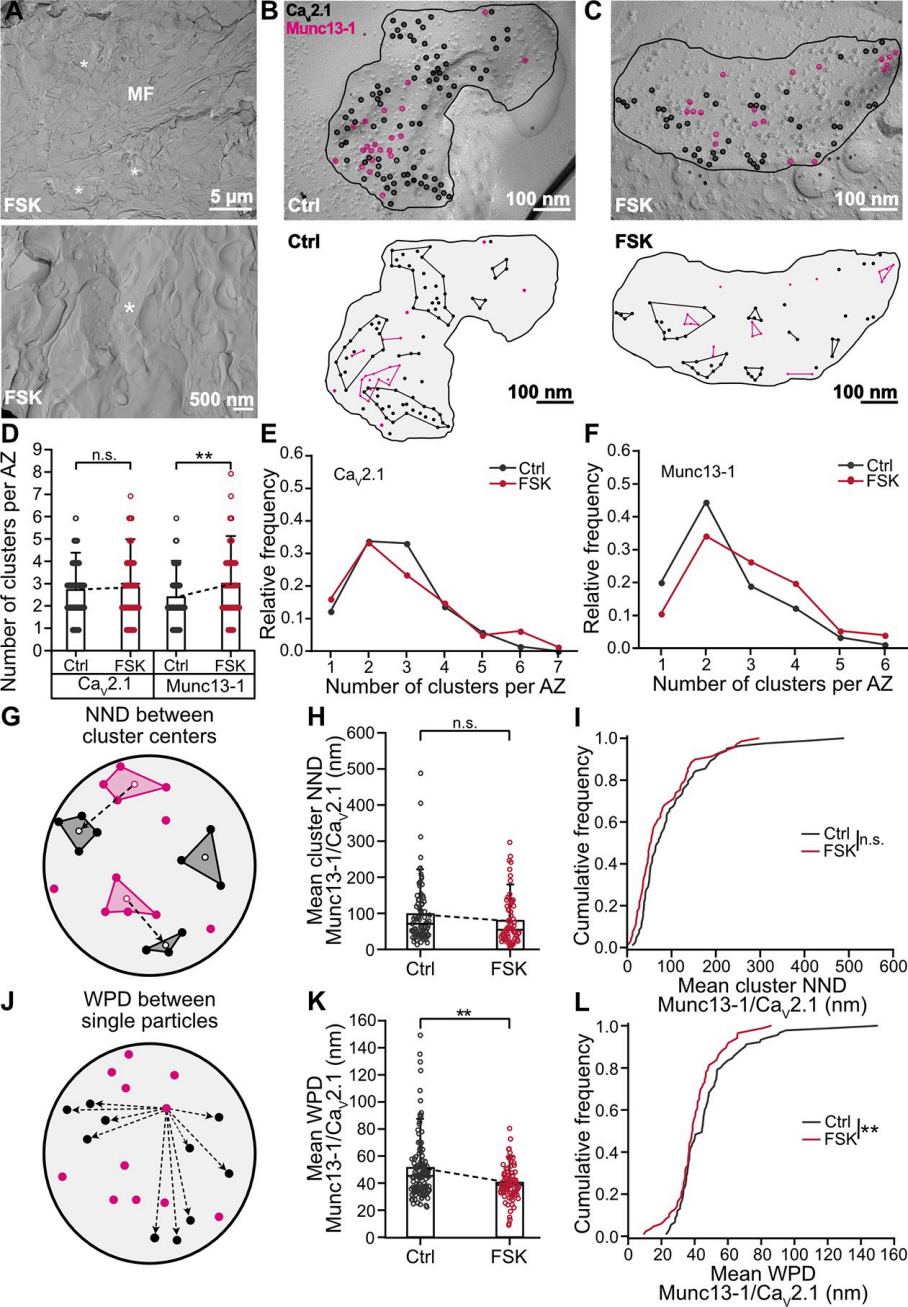
Rearrangement of Ca_V_2.1 and Munc13-1 proteins within MFB AZs after induction of chemical potentiation. **(A)** Top panel: example TEM micrograph of freeze-fractured replicas of acute slices showing ultrastructural quality of *stratum lucidum* of the CA3 region, with MF axon and putative MFBs (white asterisks) after 50 μM forskolin (“FSK”). Bottom panel: higher magnification TEM micrograph showing putative MFB (white asterisk) in stratum lucidum of CA3 region after forskolin (“FSK”). MFBs were recognized based on characteristic morphological features: large bouton size and presence of adjacent cross-fracture with high density of SVs**. (B, C)** Top panel: higher magnification micrographs with putative MFB AZs (black line) co-labeled against Ca_V_2.1 (black empty circles) and Munc13-1 (pink empty circles) in DMSO control (B, “Ctrl”) and after forskolin treatment (C, “FSK”). Bottom panel: schematic of the above AZs with Ca_V_2.1 and Munc13-1 clusters (black and pink circles connected with lines, respectively) in DMSO control (B, “Ctrl”) and after forskolin treatment (C, “FSK”). Note that some Ca_V_2.1 and Munc13-1 particles do not belong to any cluster and are considered “noise” points (single black and pink circles, respectively). **(D)** Summary bar graph of the number of Ca_V_2.1 and Munc13-1 clusters per AZ in DMSO control (“Ctrl,” gray) and after forskolin (“FSK,” red). Bars and whiskers show mean + SD. Horizontal black lines indicate median values. Ca_V_2.1: *P* = 0.1595; Munc13-1: *P* = 0.0088, both Mann–Whitney tests. **(E, F)** Relative frequency distribution of data displayed in (D), Ca_V_2.1 (E), Munc13-1 (F), color scheme is identical to (D). **(G)** Scheme of distances between Munc13-1 and Ca_V_2.1 clusters (pink and black polygons, respectively). NND between clusters was quantified as Euclidian distance (dashed line) between centers of Munc13-1 clusters (empty pink circles) to the closest center of Ca_V_2.1 cluster (empty black circles). The results are shown in (H and I). **(H)** Summary bar graph of the mean NND between Munc13-1 and Ca_V_2.1 clusters in DMSO control (“Ctrl,” gray) and after forskolin (“FSK,” red). Bars and whiskers show mean + SD. Horizontal black lines indicate median values. *P* = 0.0653, Mann–Whitney test. **(I)** Cumulative plots of mean NND between Munc13-1 and Ca_V_2.1 clusters, color scheme is identical to (H). *P* = 0.0653, Mann–Whitney test. **(J)** Scheme of WPD quantification between Munc13-1s and Ca_V_2.1 channels. WPD between particles was quantified as Euclidian distance (dashed lines) between each Munc13-1 particle (pink circles) to each Ca_V_2.1 particle (black circles). The results are shown in (K and L). **(K)** Summary bar graph of the mean WPDs between Munc13-1 and Ca_V_2.1, color scheme is identical to (H). Bars and whiskers show mean + SD. Horizontal black lines indicate median values. *P* = 0.0034, Mann–Whitney test. **(L)** Cumulative plots of mean WPDs between experimental Munc13-1 and Ca_V_2.1 point patterns, color scheme is identical to (H). *P* = 0.0034, Mann–Whitney test. Scale bar sizes are indicated on the figure panels. See S3–S9 Figs. Numerical values for this figure are detailed at https://doi.org/10.15479/AT:ISTA:18296. AZ, active zone; DMSO, dimethyl sulfoxide; MF, mossy fiber; MFB, mossy fiber bouton; NND, nearest neighbor distance; SD, standard deviation; SV, synaptic vesicle; TEM, transmission electron microscopy; WPD, weighted pairwise distance.

Forskolin may alter the molecular nanoarchitecture of MFB AZs, such as the distribution of Ca^2+^ channels or other AZ proteins in hippocampal MFB terminals [47,56,57]. To localize membrane-bound proteins with nanometer precision within the AZ, we employed FRL (Fig 4). To probe the Ca^2+^ channel–sensor coupling topography, we targeted Ca_V_2.1, the major type of presynaptic Ca^2+^ channel in MFBs [58,59] and Munc13-1/2, putative markers of primed vesicles [14,42–45,60,61]. Although Munc13s do not have transmembrane domains, Munc13-1 in synapses appears to be tightly anchored to the AZ via scaffold proteins [62,63] and interacts with components of the sub-membranous cytoskeleton [64], rendering it effectively insoluble [65]. The corresponding AZ membrane association can be detected by FRL [23,61,66,67]. Thus, after cryo-fixation, samples were fractured, and replicas were labeled with anti-Ca_V_2.1 and anti-Munc13-1 antibodies (Figs 4, S3–S6, and S9; see S1 Table). The density of the labeling of both type of particles remained the same in the presence and absence of forskolin (S4A–S4C Fig; number of particles per 0.1 μm^2^ of AZ area: Ca_V_2.1 –control: 36.2 ± 12.3 (mean ± SD), median 35.0, *n* = 130 AZs, *N* = 5 mice; forskolin: 38.8 ± 15.3, median 38.8, *n* = 52 AZs, *N* = 3 mice; Munc13-1 –control: 26.0 ± 12.5 (mean ± SD), median 23.4, *n* = 66 AZs, *N* = 3 mice; forskolin: 30.1 ± 13.4, median 28.0, *n* = 52 AZs, *N* = 3 mice; Ca_V_2.1: control versus forskolin: *P* = 0.3598; Munc13-1: control versus forskolin: *P* = 0.0689, Mann–Whitney test).

Nearest neighbor distance (NND) and modified Ripley H-function analysis revealed that Ca_V_2.1s and Munc13-1s formed clusters both in the presence and absence of forskolin, as cumulative frequency curves and H-functions of experimental data were significantly different from corresponding random distributions (S4 and S5 Figs and S1 Table). DBSCAN analysis [68] was used to determine the number of Ca_V_2.1 and Munc13-1 clusters (Figs 4, S3, and S6). The mean number of Ca_V_2.1 clusters did not change in samples cryo-fixed after forskolin treatment (Fig 4D and 4E; number of clusters–control: 2.7 ± 1.1 (mean ± SD), median 3, *n* = 139 AZs, *N* = 5 mice; forskolin: 2.8 ± 1.4, median 3, *n* = 81 AZs, *N* = 3; control versus forskolin: *P* = 0.1595, Mann–Whitney test; see S2 Table). In contrast, the number of Munc13-1 clusters increased significantly in samples cryo-fixed after forskolin application (Fig 4D and 4F; number of clusters–control: 2.4 ± 1.1 (mean ± SD), median 2, *n* = 119 AZs, *N* = 5 mice; forskolin: 3.0 ± 1.4, median 3, *n* = 78 AZs, *N* = 3 mice; control versus forskolin: *P* = 0.0088, Mann–Whitney test; see S2 Table).

The hippocampal MFB synapses are synapses with low initial P_r_ [51,69,70]. This may be explained by loose coupling between the Ca^2+^ source (Ca^2+^ channels) and the release sensor (synaptotagmin on SVs) in MFBs [71]. To investigate the structural correlates of coupling, FRL experiments with co-labeling of Munc13-1 and Ca_V_2.1 were performed. Interestingly, the mean NND between Munc13-1 and Ca_V_2.1 clusters showed a tendency for a decrease after forskolin treatment (Fig 4G–4I; S2 Table; NND–control: 97.4 ± 83.0 nm (mean ± SD), median 70.8 nm, *n* = 83 AZs, *N* = 5 mice; forskolin: 79.1 ± 67.2 nm, median 54.2 nm, *n* = 69 AZs, *N* = 3 mice; control versus forskolin: *P* = 0.0653, Mann–Whitney test). Furthermore, the mean weighted pairwise distance (WPD) significantly decreased (Fig 4J–4L and S2 Table; WPD–control: 51.2 ± 24.2 nm (mean ± SD), median 45.3 nm, *n* = 110 AZs, *N* = 5 mice; forskolin: 40.3 ± 12.8 nm, median 38.7 nm, *n* = 85 AZs, *N* = 3 mice; control versus forskolin: *P* = 0.0034, Mann–Whitney test). These results suggest that the increase in P_r_ after induction of chemical potentiation might be related to a tightening of the coupling configuration [8].

To further investigate changes in molecular architecture during chemical potentiation, we next focused on Rab3A-interacting molecule (RIM1/2) proteins (S7 Fig). RIM1/2 recruits Munc13-1s to AZs [72,73] and interacts with other AZ components to enable exocytosis [73–76]. Notably, the number of RIM1/2 clusters increases after forskolin treatment (S7D and S7E Fig; number of clusters–control: 2.3 ± 1.3 (mean ± SD), median 2, *n* = 76 AZs, *N* = 3 mice; forskolin: 3.1 ± 1.6, median 3, *n* = 62 AZs, *N* = 3 mice; control versus forskolin: *P* = 0.0042, Mann–Whitney test). However, neither NND between RIM1/2 and Ca_V_2.1 clusters nor WPD between single particles changed after forskolin (S7F–S7I Fig; NND–control: 139.5 ± 95.4 nm (mean ± SD), median 121.1 nm, *n* = 63 AZs, *N* = 3 mice; forskolin: 152.3 ± 117.3 nm, median 125.3 nm, *n* = 55 AZs, *N* = 3 mice; control versus forskolin: *P* = 0.6350, Mann–Whitney test; WPD–control: 60.2 ± 34.5 (mean ± SD), median 48.4 nm, *n* = 77 AZs, *N* = 3 mice; forskolin: 58.4 ± 25.2 nm, median 50.0 nm, *n* = 63 AZs, *N* = 3 mice; control versus forskolin: *P* = 0.7156, Mann–Whitney test).

After Munc13-1, the second-most abundant isoform expressed at MFB synapses is bMunc13-2 [40,42,77]. To test the contribution of bMunc13-2 proteins to the priming of SVs during PKA-dependent potentiation, we examined the distribution of bMunc13-2 proteins in MFB AZs (S3–S6 and S8 Figs; see S1 Table). Both NND and Ripley H-function analysis revealed a tendency of bMunc13-2 proteins to cluster within MFB AZs in the presence and absence of forskolin, albeit significant only in the case of the NND (S3–S5 Figs; see S1 Table). Unlike Munc13-1, bMunc13-2 did not show any significant alterations in the cluster number after forskolin application (S8C and S8D Fig; number of clusters–control: 1.8 ± 0.7 (mean ± SD), median 2, *n* = 60 AZs, *N* = 3 mice; forskolin: 1.8 ± 0.9, median 2, *n* = 51 AZs, *N* = 3 mice; control versus forskolin: *P* = 0.8124, Mann–Whitney test; see S2 Table). Furthermore, no change in the mean NND and the mean WPD between bMunc13-2 and Ca_V_2.1 was observed (S8E–S8H Fig; NND–control: 70.7 ± 49.2 nm (mean ± SD), median 61.8 nm, *n* = 53 AZs, *N* = 3 mice; forskolin: 73.8 ± 52.0 nm, median 62.4 nm, *n* = 44 AZs, *N* = 3 mice; control versus forskolin: *P* = 0.5943, Mann–Whitney test; WPD–control: 51.6 ± 19.2 nm (mean ± SD), median 49.0 nm, *n* = 94 AZs, *N* = 3 mice; forskolin: 52.2 ± 16.1 nm, median 49.1 nm, *n* = 60 AZs, *N* = 3 mice; WPD–control versus forskolin: *P* = 0.6687, Mann–Whitney test; see S2 Table). These results indicate that the effects of forskolin on localization of Munc13 isoforms were differential, associated with a selective increase of Munc13-1 but not bMunc13-2 clusters. This is consistent with the hypothesis that the Munc13-1 protein isoform is the major priming factor in hippocampal MFBs, involved in the expression of PKA-dependent potentiation.

### Spatial reorganization of Munc13-1 depends on PKA activity

**Fig 5:**
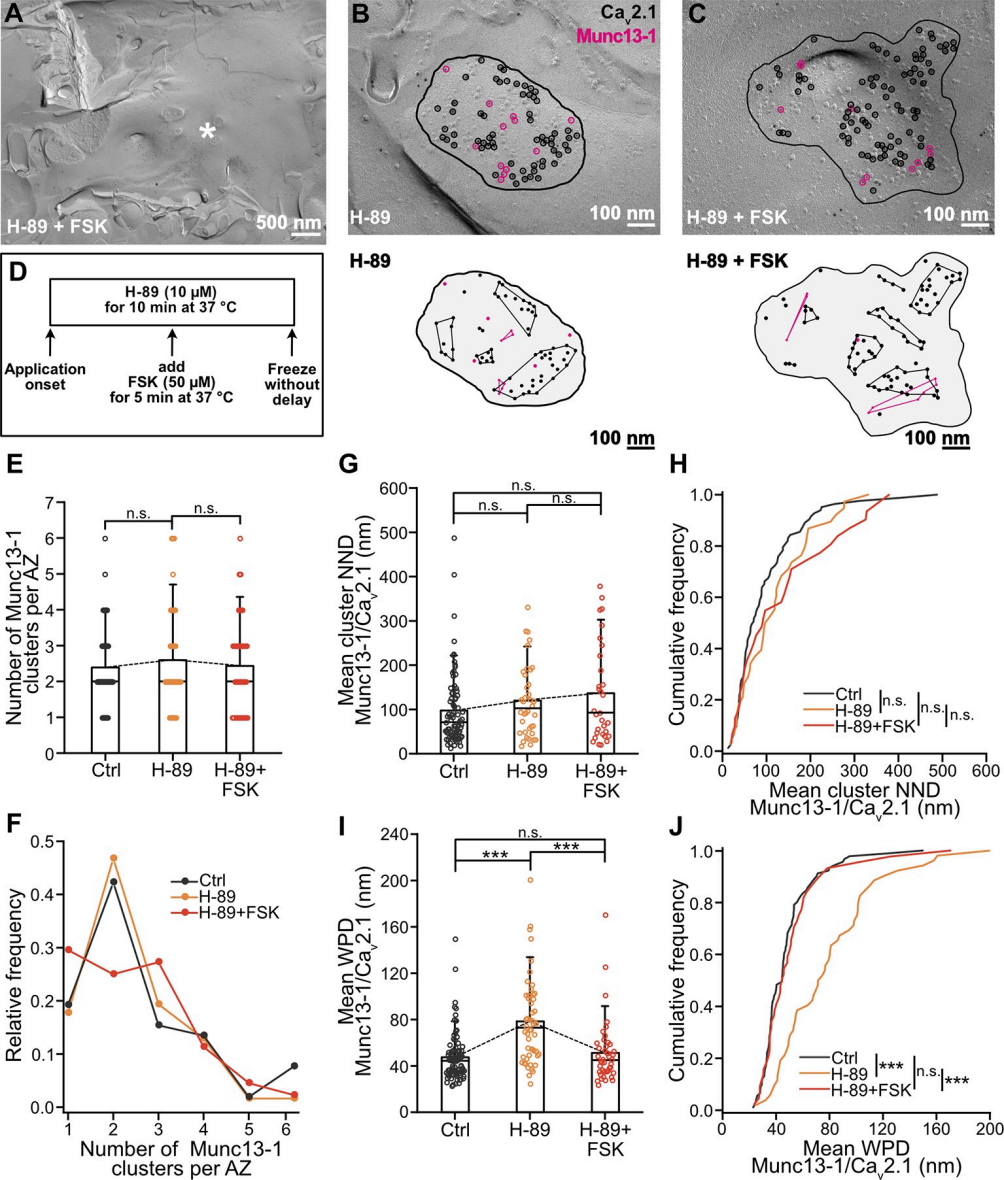
PKA-dependent remodeling of MFB AZs during chemical potentiation. **(A)** Example TEM micrograph of freeze-fractured replicas of acute slices showing putative MFB (white asterisk) after 10 μM H-89 and forskolin treatment (“H-89 + FSK”). **(B, C)** Top panel: higher magnification micrographs with putative MFB AZs (black line) co-labeled against Ca_V_2.1 (black empty circles) and Munc13-1 (pink empty circles) in H-89 treated sample (“H-89,” B) and after H-89 with FSK treatment (“H-89 + FSK,” C). Bottom panel: schematic of the above AZs with Ca_V_2.1 and Munc13-1 clusters (black and pink circles connected with lines, respectively) in H-89 treated sample (“H-89,” B) and after H-89 with FSK treatment (“H-89 + FSK,” C). Note that some Ca_V_2.1 and Munc13-1 particles do not belong to any cluster and are considered “noise” points (single black and pink circles, respectively). **(D)** Schematic representation of the time course of the experiment with 10-min H-89 and 5-min forskolin (“FSK”) treatment. **(E)** Summary bar graph of the number of Munc13-1 clusters per AZ in DMSO control (“Ctrl,” gray), after PKA inhibitor H-89 (“H-89,” orange), and H-89 with forskolin (“H-89 + FSK,” red). Bars and whiskers show mean + SD. Horizontal black lines indicate median values. Control vs. H-89: *P* = 0.6741; control vs. H-89 + forskolin: *P* = 0.9893; H-89 vs. H-89 + forskolin: *P* = 0.6869, Mann–Whitney test. **(F)** Relative frequency distribution of data shown in (E), color scheme is identical to (E). **(G)** Summary bar graph of the mean NND between Munc13-1 and Ca_V_2.1 clusters in DMSO control (“Ctrl,” gray), after H-89 (“H-89,” orange), and H-89 with FSK treatment (“H-89 + FSK,” red). Bars and whiskers show mean + SD. Horizontal black lines indicate median values. Control vs. H-89 + forskolin: *P* = 0.1430; control vs. H-89: *P* = 0.1036; H-89 vs. H-89 + forskolin: *P* = 0.8526, Mann–Whitney test. **(H)** Cumulative plots of mean NND between Munc13-1 and Ca_V_2.1 clusters, color scheme is identical to (G). Control vs. H-89 + forskolin: *P* = 0.1430; control vs. H-89: *P* = 0.1036; H-89 vs. H-89 + forskolin: *P* = 0.8526, Mann–Whitney test. **(I)** Summary bar graph of the mean WPDs between Munc13-1 and Ca_V_2.1 in DMSO control (“Ctrl,” gray), after H-89 (“H-89,” orange), and H-89 with FSK treatment (“H-89 + FSK,” red). Bars and whiskers show mean + SD. Horizontal black lines indicate median values. Control vs. H-89 + forskolin: *P* = 0.4810; control vs. H-89: *P* < 0.0001; H-89 vs. H-89 + forskolin: *P* < 0.0001, Mann–Whitney test. **(J)** Cumulative plots of mean WPDs between experimental Munc13-1 and Ca_V_2.1 point patterns, color scheme is identical to (I). Control vs. H-89 + forskolin: *P* = 0.4810; control vs. H-89: *P* < 0.0001; H-89 vs. H-89 + forskolin: *P* < 0.0001, Mann–Whitney test. Scale bar sizes are indicated in the figure panels. See S9 Fig. Numerical values for this figure are detailed at https://doi.org/10.15479/AT:ISTA:18296. AZ, active zone; DMSO, dimethyl sulfoxide; MFB, mossy fiber bouton; NND, nearest neighbor distance; SD, standard deviation; TEM, transmission electron microscopy; WPD, weighted pairwise distance.

The effects of forskolin on spatial reorganization of Munc13-1 may be PKA-dependent [29] but could also involve other pathways, e.g., Epac [78]. To test whether the changes in Munc13-1’s spatial distribution were PKA dependent, we repeated the experiments in the presence of 10 μM H-89, a PKA inhibitor (Fig 5; see S3 Table). In the presence of H-89, the number of Munc13-1 clusters per AZ did not change after forskolin application (Fig 5E and 5F; number of clusters–control: 2.4 ± 1.1 (mean ± SD), median 2, *n* = 62 AZs, *N* = 5 mice; H-89: 2.6 ± 1.4, median 2, *n* = 52 AZs, *N* = 3 mice; H-89 + forskolin: 2.4 ± 1.3, median 2, *n* = 44 AZs, *N* = 3 mice; control versus H-89: *P* = 0.6741, control versus H-89 + forskolin: *P* = 0.9893, H-89 versus H-89 + forskolin: *P* = 0.6869, Mann–Whitney test; see S3 Table). In addition, the mean NND between Munc13-1 and Ca_V_2.1 clusters was not different between experimental groups (Fig 5G and 5H; NND–control: 97.4 ± 83.0 nm (mean ± SD), median 70.8 nm, *n* = 83 AZs, *N* = 5 mice; H-89: 120.0 ± 81.4 nm, median 102.5 nm, *n* = 38 AZs, *N* = 3 mice; H-89 + forskolin: 136.5 ± 110.7 nm, median 92.8 nm, *n* = 31 AZs, *N* = 3 mice; control versus H-89 + forskolin: *P* = 0.1430; control versus H-89: *P* = 0.1036, H-89 versus H-89 + forskolin: *P* = 0.8526, Mann–Whitney test; see S3 Table). Analysis of the mean WPD between Munc13-1 and Ca_V_2.1 revealed longer WPDs in H-89 group in comparison to other 2 groups (Fig 5I and 5J; WPD–control: 47.3 ± 20.6 nm (mean ± SD), median 44.4 nm, *n* = 92 AZs, *N* = 5 mice; H-89: 78.4 ± 36.9 nm, median 73.0 nm, *n* = 52 AZs, *N* = 3 mice; H-89 + forskolin: 51.2 ± 26.9 nm, median 44.9 nm, *n* = 44 AZs, *N* = 3 mice; control versus H-89 + forskolin: *P* = 0.4810; control versus H-89: *P* < 0.0001, H-89 versus H-89 + forskolin: *P* < 0.0001, Mann–Whitney test; see S3 Table). This is consistent with a decrease in strength of synaptic transmission after H-89 application [21]. The respective change was evident in the cumulative frequency distribution of NNDs and mean WPDs between Munc13-1 and Ca_V_2.1 (Fig 5H and 5J; NND–control versus H-89: *P* = 0.1036, control versus H-89 + forskolin: *P* = 0.1430, H-89 versus H-89 + forskolin: *P* = 0.8526, Mann–Whitney test; WPD–control versus H-89 + forskolin: *P* = 0.4810; control versus H-89: *P* < 0.0001, H-89 versus H-89 + forskolin: *P* < 0.0001, Mann–Whitney test).

Application of alternative PKA inhibitor, PKI 14–22 [79] similarly blocked the increase in Munc13-1 clusters, and even decreased their number (S9A–S9D Fig; number of clusters–control: 2.4 ± 1.1 (mean ± SD), median 2, *n* = 62 AZs, *N* = 5 mice; PKI: 2.0 ± 1.0, median 2, *n* = 57 AZs, *N* = 3 mice; PKI + forskolin: 1.9 ± 1.0, median 2, *n* = 59 AZs, *N* = 3 mice; control versus PKI: *P* = 0.0514, control versus PKI + forskolin: *P* = 0.0070, PKI versus PKI + forskolin: *P* = 0.5548, Mann–Whitney test). The WPD between single Munc13-1 and Ca_V_2.1 particles lengthened in both PKI treated groups in comparison to control (S9E and S9F Fig; WPD–control: 51.2 ± 24.2 nm (mean ± SD), median 45.3 nm, *n* = 110 AZs, *N* = 5 mice; PKI: 64.9 ± 31.1 nm, median 54.0 nm, *n* = 67 AZs, *N* = 3 mice; PKI + forskolin: 66.5 ± 56.5 nm, median 49.6 nm, *n* = 67 AZs, *N* = 3 mice; control versus PKI + forskolin: *P* = 0.0009, control versus PKI: *P* < 0.0001, PKI versus PKI + forskolin: *P* = 0.2047, Mann–Whitney test). These results indicate that the forskolin-induced changes in Munc13-1’s spatial distribution are largely PKA-dependent, implying PKA-mediated changes in vesicle priming.

## Discussion

Using a combined nanophysiological and ultrastructural analysis, we examined the mechanisms underlying chemical potentiation in a hippocampal glutamatergic synapse at the level of single AZs. Functional analysis revealed that chemical potentiation increased both RRP and P_r_, by 146% and 49%, respectively. Structural analysis demonstrated that chemical potentiation increased the number of vesicles and the number of clusters of the priming protein Munc13-1 near the plasma membrane, indicating an increase in the abundance of docked and primed vesicles. Furthermore, we found a significant reorganization of the coupling configuration, including a slight reduction in NND and WPD between presynaptic Ca^2+^ channels and Munc13-1 (i.e., structural coupling). Taken together, our results provide new insights into the mechanisms of chemical potentiation at a central synapse.

### Increase in RRP and docked vesicle pool size

It is widely assumed that long-term presynaptic plasticity at hippocampal mossy fiber synapses is associated with changes in P_r_ [18,80]. In contrast, previous work on the mechanisms of PTP, a short-term form of synaptic plasticity, suggests that mainly changes in pool size are involved [21]. Our results indicate that forskolin-induced chemical potentiation also markedly increases vesicle pool size. As forskolin-induced potentiation occludes with LTP at this synapse [29], this suggests that both short-term and long-term plasticity converge on similar pool mechanisms. Thus, both short- and long-term mechanisms may involve the formation of vesicle pool engrams [21].

In addition to the biophysical analysis, our structural data may support a pool engram model. Functional analysis shows that forskolin increases the size of the RRP by 146%, i.e., to 246% of control value. Structural analysis indicates that the number of docked vesicles per 100 nm profile length changes from 0.78 to 1.20, i.e., to 154%. These one-dimensional measurements (per profile length) may need to be converted into two-dimensional predictions (per AZ area). In the extreme case, the number of docked vesicles per AZ might change to (154%)^2^ = 237%. Thus, the increase in the RRP and that in the number of docked vesicles might be in reasonable agreement. The expansion of the docked vesicle pool could be transient, as previously only a trend towards an increase in the number of docked vesicles in MFB cryo-fixed with longer delay after the forskolin application was observed [81]. RRP estimation relates to the whole bouton and it is possible that other structural changes contribute to the increase in RRP, for example an increase in release site activation, AZ area, or number [46,82].

### RRP versus docked and primed pool

The quantitative relation between functional and structural vesicle pools at central synapses has remained controversial. The RRP is often equated with the pool of docked vesicles [14] or Munc13 clusters as a proxy of primed vesicles [17,61,76] but also may represent a subpool of primed vesicles [83]. Our results shed new light on this aspect. Under control conditions, we estimate an RRP size of 1.6 vesicles per AZ (S4 Table; assuming that a typically sized MFB contains 29 AZs) [34]. This is an order of magnitude smaller than the number of docked vesicles (13.0 per AZ; S4 Table) and also less than the total number of Munc13-1/bMunc13-2 clusters (approximately 4.2). Thus, only a fraction of the docked vesicles seems to be primed and only a fraction of those is fusion-competent (1.6/4.2 = 38%). These might be “superprimed” vesicles [84,85].

In the presence of forskolin, the RRP increases to 3.8 vesicles per AZ (S4 Table). This correlates with an increased number of docked vesicles (to 21.0 per AZ) and in the number of Munc13 clusters (to 4.8 per AZ; S4 Table). After chemical potentiation, the RRP remains smaller than the number of docked and primed vesicles. This is again consistent with the existence of “superprimed” vesicles, although the fraction of these vesicles may increase (to 3.8/4.8 = 79%). A potential caveat is that the increase in the number of docked vesicles is smaller, and the increase in the number of Munc13 clusters is much smaller than the increase in the RRP. How is it possible that the number of docked and RRP vesicles markedly increases, whereas the number of Munc13 clusters increases much less? One possibility is that our FRL assay captures only a subset of primed SVs [83], as recently primed Munc13-1 proteins are not as firmly attached to the plasma membrane and might dissociate during FRL. Alternatively, it is possible that Munc13 clusters are not proxies of primed vesicles, but rather report docking/priming sites to which multiple vesicles are attached after chemical potentiation [23,45]. Consistent with this hypothesis, the number of RIM1/2 and Munc13 clusters per AZ are comparable and are increased by forskolin to roughly the same extent (S4 Table). Nonlinearities in the FRL procedure, e.g., caused by steric hindrance, need to be also considered. Finally, ubMunc13-2 or Munc13-3 could be involved in enhanced priming after chemical potentiation [40].

As the RRP does not perfectly correlate with neither docked nor primed vesicles, it may be necessary to consider additional vesicle substates, such as “tethered,” “loosely docked,” and “tightly docked” vesicles [83]. Consistent with this possibility, the RRP at mossy fiber synapses is not constant, but stimulation paradigm-dependent. Capacitance measurements from hippocampal mossy fiber terminals suggest that long depolarizing pulses release approximately 500–1,400 vesicles, corresponding to 17 to 48 vesicles per AZ [33,86]. This number is larger than that of primed vesicles and even exceeds that of docked vesicles. Thus, the long depolarizing pulse may trigger the release of tethered vesicles or, in addition, activate fast refilling of vesicles from the recycling or reserve pool [9,10].

### Changes in coupling topography during chemical potentiation

Our biophysical analysis reveals that forskolin increases P_r_, although the effects are smaller than those on the vesicular pool. Changes in both RRP and P_r_ have been also reported during PTP [21]. What is the underlying mechanism of an increase in P_r_ by forskolin? An increase in the number of Ca^2+^ channels or a functional modulation of Ca^2+^ channels may lead to larger Ca^2+^ inflow, thereby enhancing transmitter release. However, a previous study and our results show that neither of them accounts for P_r_ change upon the induction of chemical potentiation (Fig 4D and 4E) [47]. In contrast, forskolin significantly altered the coupling configuration between Ca^2+^ channels and primed vesicles. Moreover, our results revealed that baseline activity of cAMP-PKA signaling pathway is important for coupling distance, basal synaptic transmission, and the induction of presynaptic potentiation, as the application of PKA antagonists led to “uncoupling” of Munc13-1 and Ca_V_2.1 (Figs 5 and S9) and a decrease in synaptic strength [21]. Under control conditions, we found that the NND between Munc13-1/2 and Ca^2+^ channel clusters was 97.4 and 79.1 nm, whereas the mean WPD was 51.2 and 40.3 nm. This is in reasonable agreement with the biophysical coupling distance based on exogenous Ca^2+^ chelator experiments (approximately 70 nm) [71], considering effects of distant Ca^2+^ channels to transmitter release, contributions of Ca_V_2.2 (N-type) and Ca_V_2.3 (R-type) Ca^2+^ channel subtypes [58,59], and free rotation of antibodies around the epitope. However, previous studies reported a lack of change in coupling distance between Ca_V_2.1 and Munc13-1 [46,47]. There are several possible explanations for this discrepancy: (1) different imaging approaches (EM-FRL in this study versus super-resolution light microscopy (gSTED)); (2) different forskolin application times and fixation methods (5 min in our work versus 15 min; HPF versus chemical fixation); and (3) different analysis procedures (immunogold particle-based NND or WPD in the present study versus peak-to-peak fluorescence). Our results give the first direct evidence that the cAMP-PKA pathway can bidirectionally regulate the vesicle-channel topography at mossy fiber terminals, resulting in changes in P_r_.

In the presence of forskolin, the Munc13-1/ Ca_V_2.1 WPD decreased by approximately 11 nm. Can this change explain the increase in P_r_? Although the effect seems quantitatively small, the effects on transmitter release will be highly nonlinear. Modeling of Ca^2+^ domains showed that tightening of coupling increases P_r_ approximately 10-fold per 20 nm change in coupling distance [87,88]. Thus, an 11-nm decrease in the coupling distance may increase P_r_ up to 5-fold. Although forskolin shortened the distances between Munc13-1 and Ca_V_2.1, we did not observe the same effect on WPDs between RIM1/2 and Ca_V_2.1. This might result from direct, fixed-length interaction between the PZD domain of RIM1/2 and the Ca_V_2.1 C-terminus [73,74], which is difficult to modulate. In contrast, the C_2_A domain of Munc13-1 interacts with the zinc-finger domain of RIM building a heterodimeric complex [72,89–91]. The indirect interaction between Munc13-1 and Ca_V_2.1 provides a level of free movement of these proteins. Altogether, our results suggest that chemical potentiation at hippocampal mossy fiber synapses is associated with complex changes in both vesicle pool organization and coupling configuration.

### Signaling pathways and molecular targets in chemical potentiation

Forskolin increases cAMP levels by direct activation of the AC. Moreover, isoproterenol has effects comparable to forskolin, suggesting that beta 1/2 adrenoreceptors and G_S_ proteins activate the same pathway more physiologically. But what are the molecular events downstream of AC? The effects of forskolin on both the RRP and the primed vesicles are blocked by both H-89 and PKI 14–22, indicating an involvement of PKA. PKA is thought to phosphorylate several presynaptic target proteins, including synaptotagmin 12, tomosyn, rabphilins, SNAP 25 (synaptosomal-associated protein of 25 kDa), and RIMs [92]. For example, phosphorylation of SNAP 25 protein by PKA affected the size of the releasable pool in chromaffin cells [93]. Although PKA-dependent phosphorylation of RIM has been shown not to be involved in LTP [94], recent evidence highlights the importance of other phosphorylation sites of RIM during plasticity [95]. Thus, the phosphorylation of RIMs may change binding of Munc13 and thereby priming of SVs. In this context, it is interesting that forskolin changes only the localization of Munc13-1 but not bMunc13-2, which lacks C_2_A domain and is thought to prime vesicles independently of RIM [40,96]. Bassoon, a scaffold protein, is another candidate for phosphorylation, as it strongly colocalizes with Munc13-1 but not Munc13-2 at AZs [60]. This may suggest that Munc13-1 selectively translocates to the protein network of the AZ. However, contributions of other target proteins for PKA-mediated phosphorylation cannot be excluded.

### Neuromodulation of mossy fiber transmission and plasticity

Our results indicate that not only the AC activator forskolin potentiates mossy fiber synaptic transmission. In addition, the more natural agonist isoproterenol shows this effect, although its action appears to be mechanistically distinct and more variable (Fig 2). The heterogeneous expression of beta-adrenergic receptors on mossy fiber terminals, the lower level of cAMP production, and the parallel activation of G_s_ alpha and beta-gamma pathways by the natural agonist may explain the variability. Several neuromodulators, such as acetylcholine [97], dopamine [98], and noradrenaline [54] have been reported to affect mossy fiber synaptic transmission. Within the hippocampus, the *stratum lucidum* *(*the region of mossy fiber termination) is the region with the highest density of innervation by neuromodulatory inputs, such as noradrenaline, adrenaline, and dopamine [99,100]. Moreover, neuromodulators can change the induction rules and time course of mossy fiber plasticity [101,102]. However, whether neuromodulators directly affect the efficacy of synaptic transmission has remained controversial. Our results demonstrate for the first time that isoproterenol, a beta 1/2 adrenergic receptor agonist, can directly potentiate synaptic efficacy by an increase in the RRP size in a majority of MFBs at the unitary level.

### Implications for hippocampal network function

Our findings indicate that neuromodulation can regulate single-synapse computations at the hippocampal mossy fiber synapse [1,103]. The unitary EPSP recordings (Fig 2) provide direct evidence for this hypothesis, demonstrating that chemical potentiation can switch the hippocampal mossy fiber synapse from a subdetonation into a detonation mode [50,51]. Previous work showed that facilitation and PTP may lead to conditional detonation or plasticity-dependent detonation at this synapse [21,51]. Our results suggest that neuromodulation can have similar effects. Thus, burst or superburst activity in GCs [104] may lead to detonation but sparse activation of GCs in conjunction with activation of neuromodulatory pathways may also efficiently activate the CA3 network. Notably, stimulation of *locus coeruleus* neurons, presumably releasing noradrenaline or dopamine, enhances hippocampus-dependent memory [105–108]. It has been suggested that noradrenaline release from *locus coeruleus* neurons may signal arousal, novelty, and reward prediction [109]. However, the mechanisms by which this enhances memory remain unclear. Our results suggest that neuromodulator-dependent strengthening of the hippocampal teacher synapse could be involved in these complex effects [25]. Overall, the phenomenological similarities between chemically and HFS-induced potentiation suggest a common mechanism behind MFB plasticity. Future work should determine whether these presynaptic mechanisms are conserved across synapses, brain regions, and species.

## Materials and methods

### Animal experiments

Rats and mice were bred in colonies maintained in the Preclinical Facility at ISTA. All procedures strictly complied with institutional, Austrian, and European ethical regulations for animal experiments, and were approved by the Bundesministerium für Bildung, Wissenschaft und Forschung of Austria (Reference number 2020–0.648.587 and 2022–0121.440).

To determine antibody specificity against Munc13-1 and bMunc13-2, a Tg(Prox1-Cre)SJ32Gsat/Mmucd line (Mutant Mouse Resource and Research Centers) was crossed with mice in which a floxed Munc13-1 was inserted on a Munc13-2/3^(−/−)^ background [110,111]. Mice were genotyped, using DNA extracted from toe or ear clippings, to ensure homozygosity of progenies.

### Slice preparation

Acute slices were prepared from 21- to 25-day-old (P21–25) Wistar rats and 21- to 40-day-old (P21–40) C57BL/6J mice. Wistar rats were used for paired electrophysiological recordings based on previously established protocols for optimal preservation of hippocampal mossy fiber tract [112]. Mice were used for all EM and FRL experiments as an established preparation for HPF [37]. Rats or mice were lightly anesthetized with isoflurane and rapidly decapitated. The brain was dissected from the skull and a blocking “magic-cut” was performed under ice-cold high-sucrose solution containing: 64 mM NaCl for rats and 87 mM NaCl for mice experiments, 120 mM for rats and 75 mM sucrose for mice, 25 mM NaHCO_3_, 10 mM D-glucose, 2.5 mM KCl, 1.25 mM NaH_2_PO_4_, 0.5 mM CaCl_2_, and 7 mM MgCl_2_, equilibrated with 5% CO_2_/95% O_2_ gas mixture, osmolarity ~325 mOsm. Transverse hippocampal slices were sectioned at a thickness of 350 μm for paired electrophysiological recordings or 150 μm for HPF, using a vibratome (VT1200S, Leica Microsystems) in ice-cold high-sucrose solution. Slices were transferred to a maintenance chamber and recovered at 35°C for 30 to 45 min. After recovery and until use for electrophysiology recordings, slices were kept in the maintenance chamber with a high-sucrose solution at room temperature. For HPF experiments, slices were kept until freezing in a second set of maintenance chambers filled with artificial cerebrospinal fluid (ACSF) solution, identical to the solution used for electrophysiology recordings, containing: 125 mM NaCl, 25 mM D-glucose, 25 mM NaHCO_3_, 2.5 mM KCl, 1.25 mM NaH_2_PO_4_, 2 mM CaCl_2_, and 1 mM MgCl_2_, equilibrated with 5% CO_2_/95% O_2_ gas mixture.

### Paired recordings

Slices were placed in a recording chamber and superfused with ACSF for at least 15 min before onset of recording. Subcellular patch-clamp recordings from single MFBs and simultaneous recordings from CA3 PNs were performed under an upright microscope with infrared differential interference contrast as previously described [26]. Pre- and postsynaptic recording pipettes were fabricated from borosilicate glass capillaries (2.0 mm outer diameter, 1.0 mm inner diameter) and had open-tip resistances of 10 to 25 MΩ and 2 to 5 MΩ, respectively, when filled with a K^+^-based internal solution (120 mM K-gluconate, 20 mM KCl, 2 mM MgCl_2_, 2 mM Na_2_ATP, 10 mM HEPES, and 10 mM EGTA, pH adjusted to 7.3 with KOH, ~300 mOsm). The pre- and postsynaptic holding potential was set at −70 mV in the voltage-clamp configuration. APs in MFBs were evoked by brief voltage pulses (amplitude 800 to 900 mV, duration 0.1 ms) in the tight-seal, cell-attached configuration. A train stimulation (10 stimuli at 50 Hz) was delivered once every 20 s (i.e., at 0.05 Hz). Postsynaptic series resistance was 8.85 ± 0.05 MΩ (mean ± SEM; median 8.55 MΩ; ranging from 4.55 to 14.84 MΩ). Series resistance was uncompensated but carefully monitored with a test pulse (5 mV) following each data acquisition sweep. Current-clamp recordings were performed at approximately −70 mV with <150 pA injection of hyperpolarizing current. A 50-Hz train stimulation (3 pulses) was delivered once every 20 s. Membrane potential was checked repeatedly throughout the experiment, and holding current was carefully readjusted if required. In current-clamp mode, pipette capacitance was approximately 70% compensated and series resistance was balanced using the bridge circuit of the amplifier. Electrophysiology recordings were performed at approximately 24°C (22 to 25°C; Figs 1, 2, and S2) or at approximately 32°C (31 to 34°C; S1 Fig). In control condition, temperature did not affect EPSC_1_ amplitude (24°C: 154.1 ± 15.1 pA (mean ± SEM), median 146.8 pA, n = 8 cells, N = 8 rats here and below; 32°C: 437.7 ± 130.7 pA, median 465.5 pA, n = 7 cells, N = 6 rats here and below; 24°C versus 32°C: *P* = 0.3969, Mann–Whitney test) and RRP size (24°C: 1.16 ± 0.21 nA (mean ± SEM), median 1.27 nA; 32°C: 1.33 ± 0.49 nA (mean ± SEM), median 0.83 nA; 24°C versus 32°C: *P* = 0.7789, Mann–Whitney test) but did P_r_ (24°C: 0.17 ± 0.04 (mean ± SEM), median 0.12; 32°C: 0.40 ± 0.08, median 0.37; 24°C versus 32°C: *P* = 0.0014, Mann–Whitney test) and refilling rate (24°C: 15.0 ± 2.1 pA ms^−1^ (mean ± SEM), median 13.6 pA ms^−1^; 32°C: 31.2 ± 5.8 pA ms^−1^, median 24.8; 24°C versus 32°C: *P* = 0.0037, Mann–Whitney test).

### Chemical potentiation experiments

To induce chemical potentiation in MFB synapses, 50 μM forskolin (Tocris Bioscience) in 0.01% dimethyl sulfoxide (DMSO; Sigma-Aldrich) was applied to ACSF; slices were incubated for 5 min at room temperature (22 to 25°C; electrophysiology) or approximately 37°C (HPF). Acute slices were frozen immediately after forskolin application (HPF). To activate of G_s_-coupled receptors, 1 μM of isoproterenol hydrochloride (Tocris Bioscience) or (−)-isoproterenol hydrochlorid (Sigma-Aldrich) were used, slices were incubated for 10 min at room temperature (electrophysiology). During electrophysiology recordings, forskolin and isoproterenol were applied by bath application at the specified concentration. Perfusion rate was 4 to 5 ml min^−1^. During the recording of mEPSCs, 1 μM tetrodotoxin (TTX; Alomone Labs) and 10 μM gabazine (BioTrend) were added directly to ACSF.

To block PKA activity in MFB synapses, samples were incubated either in H-89-containing ACSF (10 μM in 0.01% DMSO, Tocris Bioscience) for 10 min prior to freezing or in myristoylated PKI 14-22-containing ACSF (1 μM in 0.01% DMSO, Tocris Bioscience) for 15 min prior to freezing.

### HPF of acute hippocampal slices

HPF was performed with a Leica EM ICE HPF apparatus (Leica Microsystems) as previously described [37]. Materials and samples were always kept close to physiological temperature (approximately 37°C). After slicing and recovery procedures, acute hippocampal slices were frozen with filler medium containing 15% polyvinylpyrrolidone (PVP; Sigma-Aldrich) in ACSF, equilibrated with 5% CO_2_/95% O_2_ gas mixture, and kept at 37°C. The specimen sandwich for HPF was assembled with two 120-μm-thick sapphire discs, a 150-μm-thick spacer ring, a 450-μm-thick top ring (Engineering Office M. Wohlwend, Sennwald, Switzerland), and transparent half-cylinder cartridges and middle-plate (Leica Microsystems). The outer diameter of sapphire disks and spacer rings was 6 mm. The inner diameter of the rings was either 5 mm or 4 mm depending on the size of the slice.

### Freeze-substitution and ultramicrotomy

For the freeze-substitution experiments [37], the HPF samples were put to vials with 0.1% tannic acid (Sigma-Aldrich) in acetone, transferred to a Leica AFS2, kept at −90°C, and shaken for 20 to 22 h. Next, samples were washed 3 to 4 times 10 min each with fresh acetone chilled to −90°C and left at the same temperature for 6 h in acetone containing 2% osmium (Science Services (EMS)) and 0.2% uranyl acetate (Serva). Subsequently, the temperature was raised to −60°C (10°C per hour) and was kept at −60°C for 3 h. Then, samples were heated to −30°C (10°C per hour) and kept at −30°C for 3 h; then, the temperature was finally raised to 0°C (10°C per hour). Next, the vials were washed with ice-cold acetone, 3 times for 10 min each, on ice. Then, they were washed twice with propylene oxide for 10 min each on ice and infiltrated with hard Durcupan resin (11.4 g reagent A, 10 g B, 0.3 g C, and 0.1 g D; all Sigma-Aldrich) at 2:1, 1:1, and 1:2 propylene oxide/Durcupan resin mix for at least 1 h each, shaking at room temperature. Samples were left in pure resin overnight. Samples were polymerized overnight at 100°C in BEEM capsules, and 70-nm ultrathin sections were cut with a Leica EM UC7 ultramicrotome with diamond knives (Diatome Histo). Sections were picked up on Formvar-coated copper slot grids for transmission electron microscopy (TEM) imaging. Post-staining was done with 4% uranyl acetate in water for 10 min, followed by lead citrate for 2 to 3 min (Sigma-Aldrich).

### HPF for freeze-fracture experiments

For evaluation of replicas from acute hippocampal slices, the tissue was prepared as described above with some modifications. For these experiments, 4.6-mm gold-plated carriers with 140-μm double-sided adhesive tape were used. Freeze-fracture replicas were produced using the tensile fracture approach with 2 freeze-fracture machines interchangeably: JFD V (Jeol) and ACE 900 (Leica Microsystems). Samples were fractured at −120°C under a high vacuum. Carbon-platinum replicas were prepared by evaporating layered carbon (C) at a 90° angle and platinum (Pt) at 60° on the surfaces of the slices: the first C layer was 5-nm thick, the second Pt layer was 2-nm, and the final third C layer was 20 nm. Afterwards, replicas were brought to Tris-buffered saline (TBS, 50 mM (pH 7.4)) at room temperature and transferred to glass tubes containing 2.5% sodium dodecyl sulfate (SDS) solution and 20% sucrose in 15 mM TBS (pH 8.3). Non-replicated tissue was solubilized in SDS solution for 18 h, shaking at 80°C. Next, prepared replicas were kept in the same SDS solution until immunolabeling experiments.

### Freeze-fracture replica immunolabeling (FRL)

All replicas were handled with utmost care during all steps to ensure they stayed intact. Washes were done in 12-well porcelain plates and replicas were transferred with a glass rod. First, replicas were washed with fresh 2.5% SDS in TBS solution for 10 min shaking at room temperature. Subsequently, they were transferred to 0.1% Tween in 0.05% bovine serum albumin (BSA, Sigma-Aldrich) in TBS buffer solution and washed for 10 min. Afterwards, sections were washed 3 to 4 times for 15 min in 0.05% BSA in TBS, before blocking for 90 min in 5% BSA. Then, replicas were incubated in the first primary antibody, guinea pig anti-Ca_V_2.1 (P/Q-type; Synaptic Systems, Cat # 152 205, RRID:AB_2619842, 1.3 μg ml^−1^) [113] in 2% BSA in TBS overnight, shaking at 15°C. Next, replicas were washed 3 to 4 times in 0.05% BSA in TBS for 15 min each and again blocked with 5% BSA in TBS for 90 min. Replicas were incubated in second primary antibodies, rabbit anti-Munc13-1 (Synaptic Systems, Cat #126103, RRID: AB_887733), anti-Munc13-2 (Synaptic Systems, Cat #126203, RRID: AB_2619807), or anti-RIM1/2 (Synaptic Systems, Cat # 140 208, RRID: AB_3083026; each 2.5 μg ml^−1^) overnight, shaking at 15°C. The Munc13-2 antibody was targeted against the brain-specific splice variant bMunc13-2, which is more abundant than the ubiquitous splice variant ubMunc13-2 in the hippocampus [77,96]. Then, the same washing and blocking steps were repeated. Secondary antibodies, goat anti-rabbit 5-nm gold conjugated (BBI Solutions, Cat # EM GAR5, RRID:AB_1769142) and goat anti-guinea pig 10-nm gold conjugated (BBI Solutions, Cat # EM.GAG10, RRID:AB_2892072) at concentration 1:30 in 2% BSA in TBS, were sequentially applied overnight at 15°C. Samples were picked up on Formvar-coated copper mesh grids for TEM imaging. For unequivocal distinction in double labeling experiments, secondary antibodies conjugated to gold particles of different size (5 and 10 nm) were used. Batches of secondary antibodies were tested and distribution of the particle sizes showed a clear separation between 2 populations (mean size of 5-nm antibody: (mean ± SEM) 5.07 ± 0.07 nm; 10-nm antibody: 10.12 ± 0.08 nm). The specificity of anti-Munc13-1 and anti-bMunc13-2 antibodies was confirmed using replicas of acute hippocampal slices prepared from Munc13-1cKO-Munc13-2/3^(−/−)^ animals [110,111]. The number of particles per 0.1 μm^2^ AZ of MFB from knock-out (KO) animals was compared to wild-type (WT) data from acute hippocampal slices cryo-fixed without DMSO (anti-Munc13-1 WT: 18.9 ± 1.5 (mean ± SEM), median 17.5, *n* = 40 AZs, *N* = 3 mice; cKO: 0.5 ± 0.1, median 0, *n* = 77 AZs, *N* = 3 mice; WT versus cKO: *P* < 0.0001, Mann–Whitney test; anti-bMunc13-2: WT: 15.2 ± 1.5, median 13.6, *n* = 40 AZs, *N* = 3 mice; KO: 0.5 ± 0.2, median 0, *n* = 53 AZs, *N* = 3 mice; WT versus KO: *P* < 0.0001, Mann–Whitney test).

### Electrophysiology data analysis

Data were acquired with a Multiclamp 700B amplifier, low-pass filtered at 10 kHz, and digitized at 40 or 50 kHz using a CED 1401 plus or power1401 mkII interface (Cambridge Electronic Design, Cambridge, United Kingdom). Pulse generation and data acquisition were performed using FPulse version 3.3.3 or 3.45 (U. Fröbe, Freiburg, Germany) under running Igor Pro version 6.37 (Wavemetrics). Data were analyzed with Stimfit version 0.15 and Igor Pro. Only recordings with <15 MΩ postsynaptic series resistance and stationary postsynaptic responses (EPSC_1_; based on Pearson correlation coefficient; *P* > 0.05) were included for analysis. During the 50-Hz train stimulation, the peak amplitude of each EPSC was determined as the difference between the mean of current 2-ms preceding the onset of the presynaptic voltage pulse and the mean of current over a time window ±0.5 ms around the peak. The time window of peak amplitude detection was set between the offset of voltage pulse and the next onset of voltage pulse (19.9 ms). The PPR (EPSC_2_/EPSC_1_) was calculated from average EPSC amplitudes before (10 individual traces) and after forskolin or isoproterenol application (5 or 10 individual traces).

To estimate RRP size, P_r_, and refilling rate, cumulative EPSC peak amplitudes were plotted against time for 10 stimuli at 50 Hz, the last 4 data points were fit by linear regression, and back-extrapolated to the time point 0 [49]. The size of the RRP was determined as the intersection of the regression line with the ordinate. P_r_ was measured as the ratio of EPSC_1_ amplitude over RRP size, and refilling rate was obtained from the slope of the line. Note that the RRP estimates by this method represent “pool decrement” rather than absolute pool size [11].

mEPSCs were detected using a template-fit method [69,114] running under MATLAB R2017a (MathWorks). Membrane potentials are given without correction for liquid junction potentials. In total, data reported in this paper were obtained from 35 MFB–CA3 PN paired recordings and 11 CA3 PNs.

### TEM imaging, AZ profile, and FRL analysis

All EM micrographs from ultrathin sections were analyzed blindly toward the condition of treatment; an identification number (IN) was assigned to each sample during HPF that was changed during FS or FRL. Finally, INs were randomized during sectioning, imaging, and analysis. Hippocampal MFBs were identified in the CA3b/c subregions in *stratum lucidum*. They were recognized based on previously well-characterized morphological features: large size, high density of clear SVs, presence of large dense-core vesicles, high density of mitochondria, multiple synaptic contacts with large spines, and nonsynaptic puncta adhaerentia contacts with dendritic shafts [36,37]. AZs were defined as the part of presynaptic membrane directly opposed to the electron dense region of the postsynaptic membrane (postsynaptic density), with an accumulation of clear and round vesicles in close proximity to the membrane and characteristic widening of the synaptic cleft [34,37]. Ultrastructural analysis focused on identifying the number and diameter of vesicles docked at identified AZ profiles.

Micrographs of ultrathin sections were taken with a transmission electron microscope (Thermo Fisher/FEI Tecnai 10, 80 kV acceleration voltage) with an OSIS Megaview III G3 camera and Radius acquisition software. All EM images were analyzed with Fiji open source software [115]. To optimize double membrane visualizations and accurate measurements, brightness and contrast were adjusted in Fiji. Multiple AZ profiles were analyzed per mouse, in at least 3 mice. Vesicles, whose outer membrane and presynaptic AZ membrane were in direct contact, were considered “docked.” For quantitative comparison between groups, numbers of docked vesicles per profile were normalized to 100 nm of AZ profile length.

Replicas were imaged as described for ultrathin sections. Similar to the ultrathin sections, MFBs were observed in *stratum lucidum* of the CA3b/c regions, along the mossy fiber tract. In replicas, MFBs were identified based on their location, size of the terminals, availability of attached cross-fracture with numerous SVs, and several putative AZs on the P-face of plasma membrane [39]. ImageJ Fiji open-source software was used for the analysis of all replica micrographs. AZs were recognized based on the P-face location, distribution of intramembrane particles, and labeling of AZ proteins and Ca^2+^ channels. Gold particles and AZ area were manually segmented. Their corresponding coordinates (i.e., pixel positions on an image) were used for subsequent point-pattern analysis. The number of gold particles was specified per 0.1 μm^2^ of AZ area and used as a labeling density criterion for direct comparison across groups. NND for single point pattern was calculated as a Euclidian distance from each point to its nearest neighbor. Random NND distributions were calculated from randomly distributed Poisson point patterns with densities identical to experimental values. Mean NNDs were calculated as mean value of all obtained NNDs within or between point patterns. The mean WPD was calculated as the distance from each point in the first point pattern (Munc13-1 or 2) to each point in the second point pattern (Ca^2+^ channels). Subsequently, the distances were weighted using following function [116]:

$$f(r)={\left(1/r*exp[-r/\lambda ]\right)}^{n},$$

where *r* is measured experimental distance, *n* is power coefficient (1.77) [71], *λ* is the length constant that depends on fast Ca^2+^ buffer concentration, buffer kinetics, and resting Ca^2+^ concentration:

$$\lambda =\surd (D_{Ca}/(k_{on}^{B}*B)$$

$${B=B}_{tot}*K_{D}/(K_{D}+{\left[Ca\right]}_{rest}),$$

where *D*_*Ca*_ – diffusion coefficient of free Ca^2+^, 220 μm^−2^ s^−1^; $k_{on}^{B}$ – rate constant of Ca^2+^ binding to fast exogenous buffer, 4 × 10^8^ M^−1^ s^−1^; *B*_*tot*_ – total Ca^2+^ buffer concentration, 300 μM; *B*, free Ca^2+^ buffer concentration; *K*_*D*_ – dissociation constant of exogenous buffer, 0.22 μM; *[Ca]*_*rest*_ – resting Ca^2+^ concentration, 0.074 μM [117]. To compute the weighted distance, pairwise distances from same AZs were multiplied with the corresponding weight factor and averaged. For comparison across groups, weighted distance values were pooled.

Density-based spatial clustering of applications with noise (DBSCAN) [68] was performed to determine the number of clusters of Ca^2+^ channels (Ca_V_2.1), Munc13s, and RIM1/2s. The minimum number of points per cluster was set to 2. The size of neighborhood *ε*, the distance between 2 points that can be part of the same cluster, was determined from “knee” values of 2-nearest neighbor plots (K-plots) for each analyzed AZ. Most *ε* values were in the range of mean ± 2 SD of NND. The mean *ε* values were 37.2 nm (Ca_V_2.1 control), 37.6 nm (Ca_V_2.1 forskolin), 41.3 nm (Munc13-1 control), 42.2 nm (Munc13-1 forskolin), 54.3 nm (bMunc13-2 control), 51.2 nm (bMunc13-2 forskolin), 50.8 nm (RIM1/2 control), and 62.0 nm (RIM1/2 forskolin). The intercluster distance was determined as minimal distances between cluster edges. NND between clusters of 2 distinct point patterns within the same AZ was calculated as Euclidean distance from the center of each cluster in the first point pattern (Munc13-1 or 2, RIM1/2) to the nearest cluster center in the second point pattern (Ca^2+^ channels). The analysis of NND and clustering was done in R (RStudio) using spatstat (2.0–1) and dbscan (1.1–8) CRAN packages. The weighting of pairwise distances was done in MATLAB R2017a (MathWorks).

Univariate Ripley K-function [118] and corrected H-function [119] for Ca_V_2.1 and Munc13-1/2 particles were computed using a Matlab software package. Mean experimental H-function was compared to mean simulated H-function using a maximum absolute difference (MAD) test. Mean simulated H-function was estimated from the population of all Monte-Carlo simulated H-functions (100 for each AZ).

### Statistical analysis

Statistical analysis was performed with Origin 2019 (Origin Lab), Prism (GraphPad), and RStudio. All electrophysiology data was tested using two-sided paired nonparametric Wilcoxon signed-rank, two-sided unpaired nonparametric Mann–Whitney test, or two-sided paired *t* test. All EM data groups were tested for normality with the D’Agostino-Pearson test, followed by a nonparametric Kruskal–Wallis test as appropriate. Data groups were then compared using two-sided unpaired nonparametric Mann–Whitney test. For the graphical representation of statistics, * indicates *P* < 0.05, ** *P* < 0.01, *** *P* < 0.001, and **** *P* < 0.0001. In text and figures, values report mean and median, and errors report standard error of the mean (SEM; electrophysiology) or standard deviation (SD; EM and FRL), as specifically stated.

## Supporting information

S1 Fig Related to Fig 1. The temperature dependence of synaptic transmission at MFB–CA3 PN synapses. **(A)** Top left panel: 50-Hz train of 10 stimuli. Bottom left panel: average excitatory postsynaptic current (EPSC) at 24°C. Top right panel: 50-Hz train of 10 stimuli. Bottom right panel: average EPSC at 32°C (orange). **(B)** Summary bar graph of EPSC_1_ peak amplitudes at 24°C (black) and 32°C (orange). Bars and whiskers show mean + SEM; *P* = 0.3969, Mann–Whitney test. Data from 8 cells in 8 rats (24°C) and 7 cells in 6 rats (32°C). **(C)** Cumulative plot of EPSC peak amplitudes during a 50-Hz train with 10 stimuli at 24°C (black) and 32°C (orange). Data points during the last 4 stimuli (at time points ≥120 ms) were fit by linear regression and back-extrapolated to time point 0. **(D–F)** Summary bar graphs of readily RRP (D; *P* = 0.7789), P_r_ (E; *P* = 0.0014), and refilling rate (F; *P* = 0.0037, Mann–Whitney tests), estimated from the cumulative EPSC plot (C), at 24°C (black) and 32°C (orange). Bars and whiskers show mean + SEM. Numerical values for this figure are detailed at https://doi.org/10.15479/AT:ISTA:18296.
(TIF)

S2 Fig Related to Fig 1. The effect of forskolin on miniature EPSCs in CA3 PNs. **(A)** Schematic representation of time course of the experiment; 1 μM TTX and 10 μM gabazine were added to ACSF and perfused for at least 10 min prior to the onset of recordings. Control data (“Ctrl,” gray) was recorded for 5 min prior to forskolin application. Forskolin data was recorded during last 5 min of 10-min forskolin treatment (“FSK,” red). **(B)** Representative traces of mEPSCs before (“Ctrl,” gray) and after 50 μM forskolin (“FSK,” red). **(C)** mEPSCs at expanded time scale after detection and alignment to the onset time point before (“Ctrl,” gray) and after 50 μM forskolin (“FSK,” red). Black line represents average. **(D)** Histogram of mEPSC frequency before (“Ctrl,” gray) and after 50 μM forskolin (“FSK,” red). Data from 11 cells and 3 rats. **(E)** Summary bar graph of mEPSC frequencies before (“Ctrl,” gray) and after 50 μM forskolin (“FSK,” red). Bars and whiskers show mean + SEM. *P* = 0.0185, Wilcoxon signed-rank test. **(F)** Histogram of mEPSC peak amplitude before and after 50 μM forskolin, color scheme is identical to (D). Data from 11 cells and 3 rats. **(G)** Summary bar graph of mEPSC peak before (“Ctrl,” gray) and after 50 μM forskolin (“FSK,” red). Bars and whiskers show mean + SEM. *P* = 0.4131, Wilcoxon signed-rank test. Numerical values for this figure are detailed at https://doi.org/10.15479/AT:ISTA:18296.
(TIF)

S3 Fig Related to Fig 4. Labeling of Munc13s in MFBs in wild-type and knock-out mice. **(A)** Example TEM micrographs of freeze-fractured replicas of acute wild-type (“WT”) slices. Left panel: putative MFB AZ with gold particles of 2 sizes, 10 and 5 nm. Right panel: putative MFB AZ (black line) co-labeled against Ca_V_2.1 (black dots) and Munc13-1 (pink empty circles). **(B)** Example TEM micrograph of freeze-fractured replicas of acute slices from floxed Munc13-1-Prox1Cre mice (“cKO”) showing putative MFB AZ (black line) co-labeled against Ca_V_2.1 (black dots) and Munc13-1 (pink empty circle). **(C)** Example TEM micrograph of freeze-fractured replicas of acute slices showing putative MFB AZ (black line) labeled only against Munc13-1 (black dots). **(D)** Example TEM micrographs of freeze-fractured replicas of acute wild-type (“WT”) slices. Left panel: putative MFB AZ with 10- and 5-nm gold particles. Right panel: putative MFB AZ (black line) co-labeled against Ca_V_2.1 (black dots) and bMunc13-2 (pink empty circles). **(E)** Example TEM micrograph of freeze-fractured replicas of acute slices from Munc13-2/3^(−/−)^ mice (“KO”) showing putative MFB AZ (black line) co-labeled against Ca_V_2.1 (black dots) and bMunc13-2 (pink empty circles). **(F)** Example TEM micrograph of freeze-fractured replicas of acute slices showing putative MFB AZ (black line) labeled only against bMunc13-2 (black dots). **(G, H)** Summary bar graph of the number of Munc13-1 (G) and bMunc13-2 (H) particles per 0.1 μm^2^ of AZ area in WT control (“WT,” black) and in floxed Munc13-1-Prox1Cre mice (“cKO,” orange) and Munc13-2/3^(−/−)^ mice (“KO,” orange). Bars and whiskers show mean + SD. Horizontal black lines indicate median values. Munc13-1: WT vs. cKO: *P* < 0.0001; bMunc13-2: WT vs. KO: *P* < 0.0001, both Mann–Whitney tests. **(I, J)** Summary bar graph of the number of Munc13-1 (I) and bMunc13-2 (J) particles per 0.1 μm^2^ of AZ area in single (“SL”) and double (“DL”) labeling experiments. Bars and whiskers show mean + SD. Horizontal black lines indicate median values. Number of particles per 0.1 μm^2^ of AZ area in SL–Munc13-1: 17.4 ± 8.2 (mean ± SD), median 15.8, *n* = 53 AZs, *N* = 3 mice; bMunc13-2: 17.1 ± 9.1 (mean ± SD), median 15.0, *n* = 36 AZs, *N* = 3 mice; Munc13-1: DL vs. SL: Munc13-1: *P* = 0.3805; bMunc13-2: DL vs. SL: *P* = 0.3545, both Mann–Whitney tests. **(K, L)** Summary bar graph of the number Munc13-1 (K) and bMunc13-2 (L) clusters per AZ single (“SL”) and double (“DL”) labeling experiments. Bars and whiskers show mean + SD. Horizontal black lines indicate median values. Number of clusters SL–Munc13-1: 2.0 ± 1.0 (mean ± SD), median 2, *n* = 53 AZs, *N* = 3 mice; bMunc13-2: 1.6 ± 0.8 (mean ± SD), median 1, *n* = 35 AZs, *N* = 3 mice; DL vs. SL Munc13-1: *P* = 0.0510; bMunc13-2: *P* = 0.0853 both Mann–Whitney test. Scale bar sizes are indicated on the figure panels. Numerical values for this figure are detailed at https://doi.org/10.15479/AT:ISTA:18296.
(TIF)

S4 Fig Related to Fig 4. Labeling of Ca_V_2.1s and Munc13s in MFBs before and after 5-min forskolin treatment. **(A–C)** Summary bar graph of the number of Ca_V_2.1 (A; *P* = 0.3598), Munc13-1 (B; *P* = 0.0689), and bMunc13-2 (C; *P* = 0.9543, Mann–Whitney tests) particles per 0.1 μm^2^ of AZ area in DMSO control (“Ctrl,” gray) and after 50 μM forskolin (“FSK,” red). Bars and whiskers show mean + SD. Horizontal black lines indicate median values. **(D–F)** Cumulative plots of mean NND between experimental Ca_V_2.1 (D; *P* < 0.0001), Munc13-1 (E; *P* < 0.0001), and bMunc13-2 (F; *P* < 0.0001, Mann–Whitney tests) point patterns and randomly simulated data in treated groups. Experimental data DMSO control (“Ctrl,” dark gray) and after forskolin (“FSK,” dark red), randomly simulated data DMSO control (“Ctrl *Null*,*”* light gray) and after forskolin (“FSK *Null*,*”* light pink). Numerical values for this figure are detailed at https://doi.org/10.15479/AT:ISTA:18296.
(TIF)

S5 Fig Related to Fig 4. Univariate Ripley-function of Ca_V_2.1s and Munc13s proteins particles in MFBs before and after 5-min forskolin treatment. **(A, B)** Univariate H(r) function of Ca_V_2.1 in DMSO control (A) and after 50 μM forskolin (B); *n* AZ = 90 and 81, respectively, total *P*-value on the figure. Red line indicates population mean, black line random distribution, and gray area—confidence envelopes (CE). Inset: pie chart of statistical significance of MAD test of single AZs. **(C, D)** Univariate H(r) function of Munc13-1 in DMSO control (C) and after 50 μM forskolin (D); *n* AZ = 90 and 81, respectively, total *P*-value on the figure. Red line indicates population mean, black line–random distribution, and gray area–confidence envelopes (CE). Inset: pie chart of statistical significance of MAD test of single AZs. **(E, F)** Univariate H(r) function of bMunc13-2 in DMSO control (E) and after 50 μM forskolin (F); *n* AZ = 60 and 44, respectively, total *P*-value on the figure. Red line indicates population mean, black line–random distribution, and gray area–confidence envelopes (CE). Inset: pie chart of statistical significance of MAD test of single AZs. Numerical values for this figure are detailed at https://doi.org/10.15479/AT:ISTA:18296.
(TIF)

S6 Fig Related to Fig 4. Characteristics of Ca_V_2.1 and Munc13 clusters before and after 5-min FSK application. **(A–C)** Histograms of relative frequency distribution of number of particles per Ca_V_2.1 (A), Munc13-1 (B), and bMunc13-2 (C) cluster in DMSO control (“Ctrl,” gray) and after 50 μM forskolin (“FSK,” red). Total *P*-values are indicated on the figures. **(D–F)** Histograms of relative frequency distribution of minimal distance between Ca_V_2.1 (D), Munc13-1 (E), and bMunc13-2 (F) clusters, color scheme is identical to (A–C). Total *P*-values are indicated on the figures. **(G–I)** Histograms of relative frequency distribution of area of each cluster of Ca_V_2.1 (G), Munc13-1 (H), and bMunc13-2 (I) particles, color scheme is identical to (A–C). Total *P*-values are indicated on the figures.
(TIF)

S7 Fig Related to Fig 4. Number of RIM1/2 clusters but not distance to Ca_V_2.1 channels changes during chemical potentiation. **(A)** Schematic representation of the time course of the experiment with 5-min forskolin (FSK) treatment. **(B, C)** Example TEM micrographs of freeze-fractured replica of acute slices showing putative MFB AZ (black line) co-labeled against Ca_V_2.1 (black empty circles) and RIM1/2 (yellow empty circles) in DMSO control (B, “Ctrl”) and after 50 μM forskolin (C, “FSK”). Scale bar sizes are indicated on the figure panels. **(D)** Summary bar graph of the number of RIM1/2 clusters per AZ in DMSO control (“Ctrl,” gray) and after forskolin treatment (“FSK,” red). Bars and whiskers show mean + SD. Horizontal black lines indicate median values. *P* = 0.0042, Mann–Whitney test. **(E)** Relative frequency distribution of data shown in (D), color scheme is identical to (D). **(F)** Summary bar graph of the mean NNDs between RIM1/2 and Ca_V_2.1 clusters in DMSO control (“Ctrl,” gray) and after forskolin treatment (“FSK,” red). Bars and whiskers show mean + SD. Horizontal black lines indicate median values. *P* = 0.6350, Mann–Whitney test. **(G)** Cumulative plots of mean NNDs between RIM1/2 and Ca_V_2.1 clusters, color scheme is identical to (F). *P* = 0.6350, Mann–Whitney test. **(H)** Summary bar graph of the mean WPDs between RIM1/2 and Ca_V_2.1 in DMSO control (“Ctrl,” gray) and after forskolin treatment (“FSK,” red). Bars and whiskers show mean + SD. Horizontal black lines indicate median values. *P* = 0.7156, Mann–Whitney test. **(I)** Cumulative plots of mean WPDs between experimental RIM1/2 and Ca_V_2.1 point patterns, color scheme is identical to (H). *P* = 0.7156, Mann–Whitney test. Numerical values for this figure are detailed at https://doi.org/10.15479/AT:ISTA:18296.
(TIF)

S8 Fig Related to Fig 4. bMunc13-2 shows no alterations in distribution during chemical potentiation. **(A, B)** Example TEM micrographs of freeze-fractured replica of acute slices showing putative MFB AZ (black line) co-labeled against Ca_V_2.1 (black empty circles) and bMunc13-2 (pink empty circles) in DMSO control (A, “Ctrl”) and after 50 μM forskolin (B, “FSK”). Scale bar sizes are indicated on the figure panels. **(C)** Summary bar graph of the number of bMunc13-2 clusters per AZ in DMSO control (“Ctrl,” gray) and after forskolin (“FSK,” red). Bars and whiskers show mean + SD. Horizontal black lines indicate median values. *P* = 0.8124, Mann–Whitney test. **(D)** Relative frequency distribution of data displayed in (C), color scheme is identical to (C). **(E)** Summary bar graph of the mean NNDs between bMunc13-2 and Ca_V_2.1 clusters in DMSO control (“Ctrl,” gray) and after FSK treatment (“FSK,” red). Bars and whiskers show mean + SD. Horizontal black lines indicate median values. *P* = 0.5943, Mann–Whitney test. **(F)** Cumulative plots of mean NNDs between experimental bMunc13-2 and Ca_V_2.1 clusters, color scheme is identical to (E). *P* = 0.5943, Mann–Whitney test. **(G)** Summary bar graph of the mean WPDs between bMunc13-2 and Ca_V_2.1 in DMSO control (“Ctrl,” gray) and after FSK treatment (“FSK,” red). Bars and whiskers show mean + SD. Horizontal black lines indicate median values. *P* = 0.6687, Mann–Whitney test. **(H)** Cumulative plots of mean WPDs between experimental bMunc13-2 and Ca_V_2.1 point patterns, color scheme is identical to (G). *P* = 0.6687, Mann–Whitney test. Numerical values for this figure are detailed at https://doi.org/10.15479/AT:ISTA:18296.
(TIF)

S9 Fig Related to Fig 4. Alternative PKA antagonist blocks increase in the number of Munc13-1 clusters and shortening of WPD between Munc13-1 and Ca_V_2.1. **(A)** Schematic representation of the time course of the experiment with 15-min PKI and 5-min forskolin (FSK) treatment. **(B, C)** Example TEM micrographs of freeze-fractured replica of acute slices showing putative MFB AZ (black line) co-labeled against Ca_V_2.1 (black empty circles) and Munc13-1 (pink empty circles) in sample treated with 1 μM PKI (“PKI,” B) and after PKI with FSK treatment (“PKI+FSK,” C). Scale bar sizes are indicated on the figure panels. **(D)** Summary bar graph of the number of Munc13-1 clusters per AZ in DMSO control (“Ctrl,” gray) and after PKA inhibitor PKI (“PKI,” orange), and PKI with forskolin (“PKI+FSK,” red). Bars and whiskers show mean + SD. Horizontal black lines indicate median values. Control vs. PKI: *P* = 0.0514, control vs. PKI+forskolin: *P* = 0.0070, PKI vs. PKI + forskolin: *P* = 0.5548, Mann–Whitney test. **(E)** Summary bar graph of the mean WPDs between Munc13-1 and Ca_V_2.1 in DMSO control (“Ctrl,” gray) and after PKA inhibitor PKI (“PKI,” orange), and PKI with forskolin (“PKI+FSK,” red). Bars and whiskers show mean + SD. Horizontal black lines indicate median values. Control vs. PKI + forskolin: *P* = 0.0009, control vs. PKI: *P* < 0.0001, PKI vs. PKI + forskolin: *P* = 0.2047, Mann–Whitney test. **(F)** Cumulative plots of mean WPDs between experimental Munc13-1 and Ca_V_2.1 point patterns, color scheme is identical to (E). Control vs. PKI + forskolin: *P* = 0.0154, control vs. PKI: *P* = 0.0002, PKI vs. PKI + forskolin: *P* = 0.2047, Mann–Whitney test.
(TIF)

S1 Table Related to Fig 4. Nonrandom distribution of Ca_V_2.1, Munc13-1, and bMunc13-2 proteins before and after 5-min forskolin treatment. (PDF)

S2 Table Related to Fig 4. Spatial distribution of Ca_V_2.1, Munc13-1, and bMunc13-2 proteins after 5-min forskolin treatment. (PDF)

S3 Table Related to Fig 5. Spatial distribution of Munc13-1 after treatment with the PKA inhibitor H-89. (PDF)

S4 Table Related to Figs 1–4. Comparison of RRP SV size, docked SV pool size, and number of primed SVs at hippocampal MFBs in control and during chemical potentiation. (PDF)

## Abbreviations

AC: adenylyl cyclase
ACSF: artificial cerebrospinal fluid
AP: action potential
AZ: active zone
BSA: bovine serum albumin
cAMP: cyclic adenosine monophosphate
DMSO: dimethyl sulfoxide
EM: electron microscopy
EPSC: excitatory postsynaptic current
EPSP: excitatory postsynaptic potential
FRL: freeze-fracture replica labeling
GC: granule cell
HPF: high-pressure freezing
HFS: high-frequency stimulation
IN: identification number
KO: knock-out
LTP: long-term potentiation
MAD: maximum absolute difference
MFB: mossy fiber bouton
NND: nearest neighbor distance
PN: pyramidal neuron
PPR: paired-pulse ratio
PTP: post-tetanic potentiation
RRP: readily releasable pool
SD: standard deviation
SDS: sodium dodecyl sulfate
SEM: standard error of the mean
SV: synaptic vesicle
TEM: transmission electron microscopy
WPD: weighted pairwise distance
WT: wild-type
