# Supplementary figures and images for: Presynaptic cAMP-PKA-mediated potentiation induces reconfiguration of synaptic vesicle pools and channel-vesicle coupling at hippocampal mossy fiber boutons
Source: PLoS Biol. 2024 Nov 18;22(11):e3002879. doi: 10.1371/journal.pbio.3002879 (PMC11573138; doi:10.1371/journal.pbio.3002879)

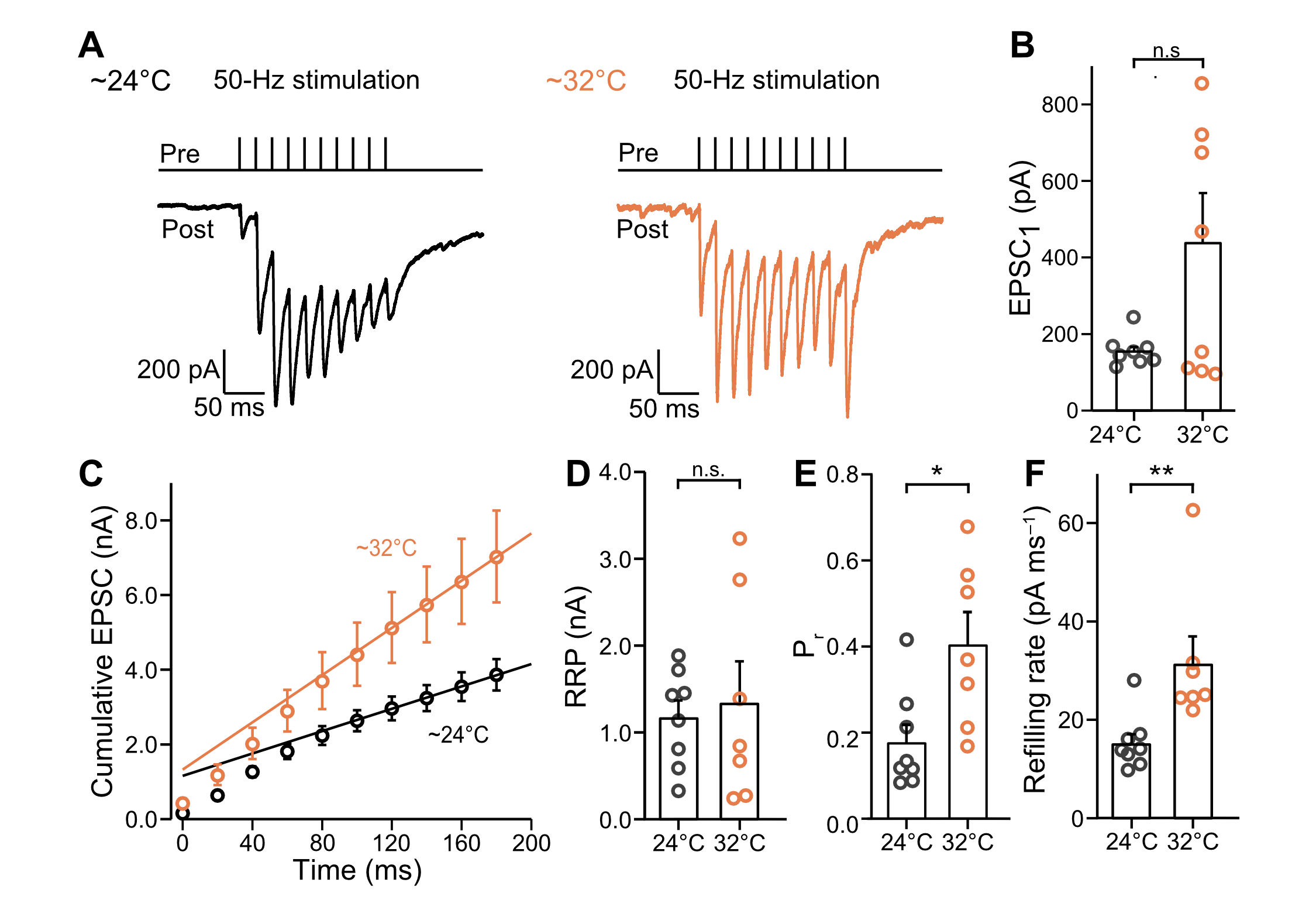

Supplement: S1 Fig — (A) Top left panel: 50-Hz train of 10 stimuli. Bottom left panel: average excitatory postsynaptic current (EPSC) at 24°C. Top right panel: 50-Hz train of 10 stimuli. Bottom right panel: average EPSC at 32°C (orange). (B) Summary bar graph of EPSC1 peak amplitudes at 24°C (black) and 32°C (orange). Bars and whiskers show mean + SEM; P = 0.3969, Mann–Whitney test. Data from 8 cells in 8 rats (24°C) and 7 cells in 6 rats (32°C). (C) Cumulative plot of EPSC peak amplitudes during a 50-Hz train with 10 stimuli at 24°C (black) and 32°C (orange). Data points during the last 4 stimuli (at time points ≥120 ms) were fit by linear regression and back-extrapolated to time point 0. (D–F) Summary bar graphs of readily RRP (D; P = 0.7789), Pr (E; P = 0.0014), and refilling rate (F; P = 0.0037, Mann–Whitney tests), estimated from the cumulative EPSC plot (C), at 24°C (black) and 32°C (orange). Bars and whiskers show mean + SEM. Numerical values for this figure are detailed at https://doi.org/10.15479/AT:ISTA:18296. (TIF) [file pbio.3002879.s001.tif]

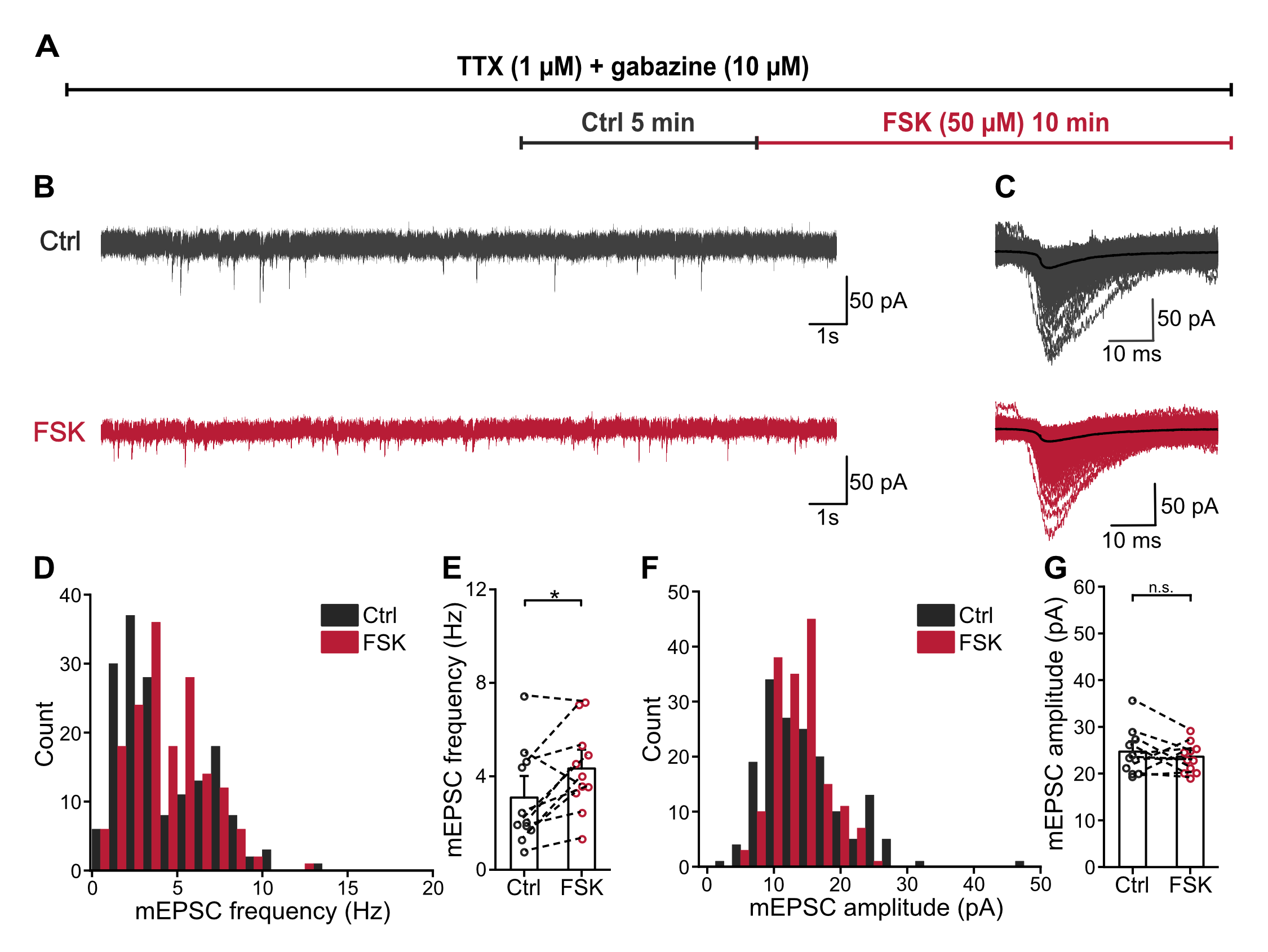

Supplement: S2 Fig — (A) Schematic representation of time course of the experiment; 1 μM TTX and 10 μM gabazine were added to ACSF and perfused for at least 10 min prior to the onset of recordings. Control data (“Ctrl,” gray) was recorded for 5 min prior to forskolin application. Forskolin data was recorded during last 5 min of 10-min forskolin treatment (“FSK,” red). (B) Representative traces of mEPSCs before (“Ctrl,” gray) and after 50 μM forskolin (“FSK,” red). (C) mEPSCs at expanded time scale after detection and alignment to the onset time point before (“Ctrl,” gray) and after 50 μM forskolin (“FSK,” red). Black line represents average. (D) Histogram of mEPSC frequency before (“Ctrl,” gray) and after 50 μM forskolin (“FSK,” red). Data from 11 cells and 3 rats. (E) Summary bar graph of mEPSC frequencies before (“Ctrl,” gray) and after 50 μM forskolin (“FSK,” red). Bars and whiskers show mean + SEM. P = 0.0185, Wilcoxon signed-rank test. (F) Histogram of mEPSC peak amplitude before and after 50 μM forskolin, color scheme is identical to (D). Data from 11 cells and 3 rats. (G) Summary bar graph of mEPSC peak before (“Ctrl,” gray) and after 50 μM forskolin (“FSK,” red). Bars and whiskers show mean + SEM. P = 0.4131, Wilcoxon signed-rank test. Numerical values for this figure are detailed at https://doi.org/10.15479/AT:ISTA:18296. (TIF) [file pbio.3002879.s002.tif]

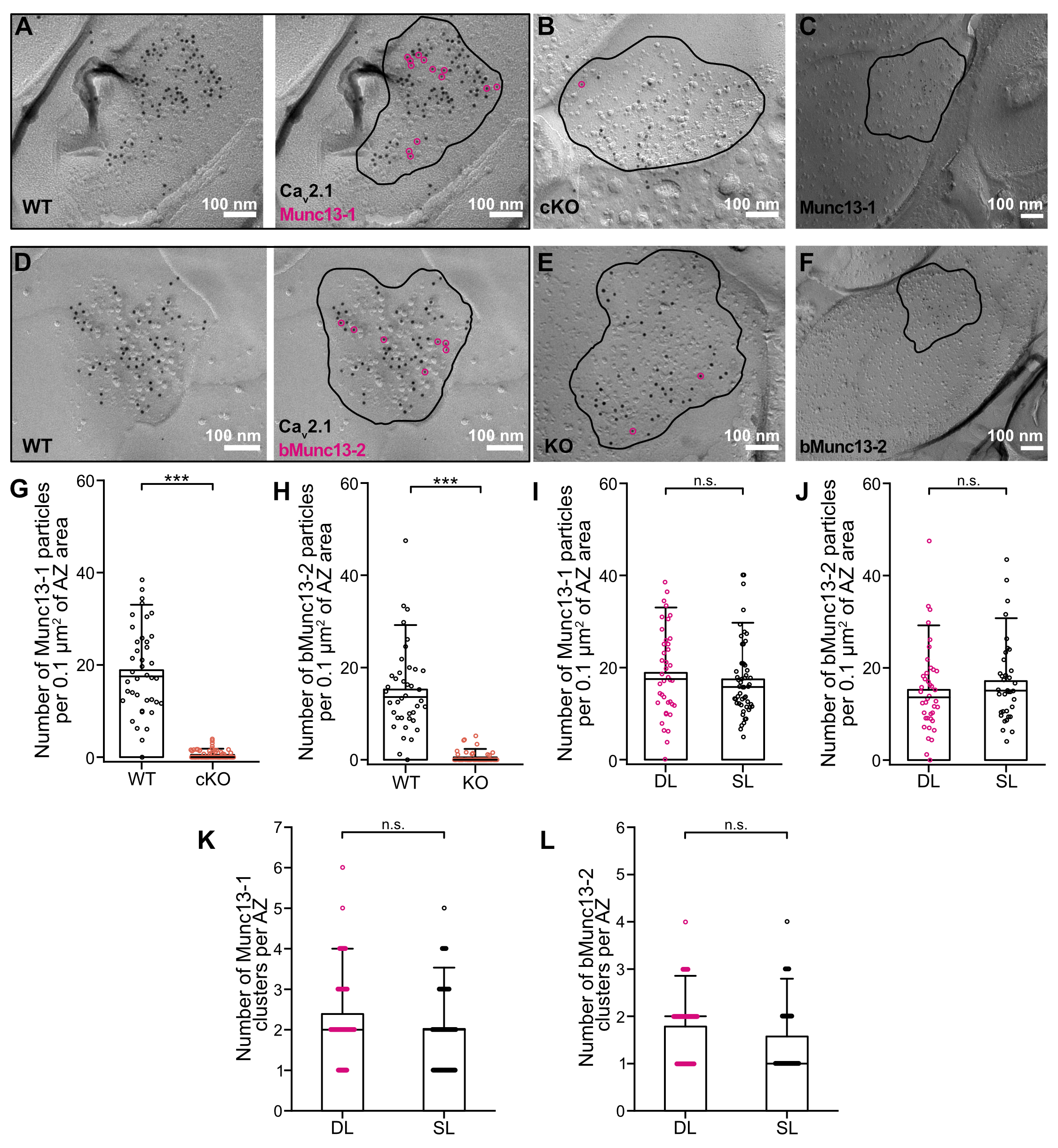

Supplement: S3 Fig — (A) Example TEM micrographs of freeze-fractured replicas of acute wild-type (“WT”) slices. Left panel: putative MFB AZ with gold particles of 2 sizes, 10 and 5 nm. Right panel: putative MFB AZ (black line) co-labeled against CaV2.1 (black dots) and Munc13-1 (pink empty circles). (B) Example TEM micrograph of freeze-fractured replicas of acute slices from floxed Munc13-1-Prox1Cre mice (“cKO”) showing putative MFB AZ (black line) co-labeled against CaV2.1 (black dots) and Munc13-1 (pink empty circle). (C) Example TEM micrograph of freeze-fractured replicas of acute slices showing putative MFB AZ (black line) labeled only against Munc13-1 (black dots). (D) Example TEM micrographs of freeze-fractured replicas of acute wild-type (“WT”) slices. Left panel: putative MFB AZ with 10- and 5-nm gold particles. Right panel: putative MFB AZ (black line) co-labeled against CaV2.1 (black dots) and bMunc13-2 (pink empty circles). (E) Example TEM micrograph of freeze-fractured replicas of acute slices from Munc13-2/3(−/−) mice (“KO”) showing putative MFB AZ (black line) co-labeled against CaV2.1 (black dots) and bMunc13-2 (pink empty circles). (F) Example TEM micrograph of freeze-fractured replicas of acute slices showing putative MFB AZ (black line) labeled only against bMunc13-2 (black dots). (G, H) Summary bar graph of the number of Munc13-1 (G) and bMunc13-2 (H) particles per 0.1 μm2 of AZ area in WT control (“WT,” black) and in floxed Munc13-1-Prox1Cre mice (“cKO,” orange) and Munc13-2/3(−/−) mice (“KO,” orange). Bars and whiskers show mean + SD. Horizontal black lines indicate median values. Munc13-1: WT vs. cKO: P < 0.0001; bMunc13-2: WT vs. KO: P < 0.0001, both Mann–Whitney tests. (I, J) Summary bar graph of the number of Munc13-1 (I) and bMunc13-2 (J) particles per 0.1 μm2 of AZ area in single (“SL”) and double (“DL”) labeling experiments. Bars and whiskers show mean + SD. Horizontal black lines indicate median values. Number of particles per 0.1 μm2 of AZ area in SL–Munc13 [file pbio.3002879.s003.tif]

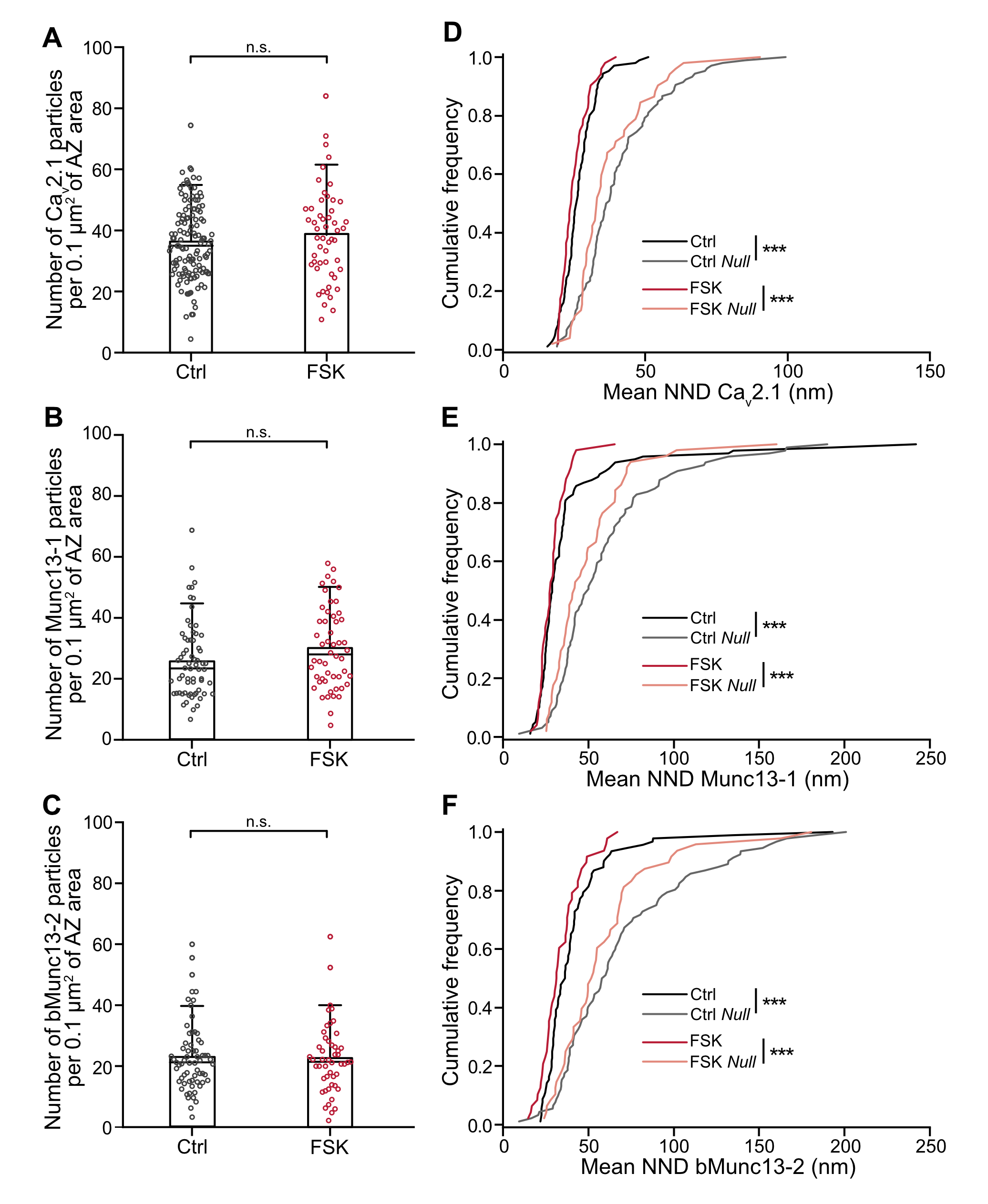

Supplement: S4 Fig — (A–C) Summary bar graph of the number of CaV2.1 (A; P = 0.3598), Munc13-1 (B; P = 0.0689), and bMunc13-2 (C; P = 0.9543, Mann–Whitney tests) particles per 0.1 μm2 of AZ area in DMSO control (“Ctrl,” gray) and after 50 μM forskolin (“FSK,” red). Bars and whiskers show mean + SD. Horizontal black lines indicate median values. (D–F) Cumulative plots of mean NND between experimental CaV2.1 (D; P < 0.0001), Munc13-1 (E; P < 0.0001), and bMunc13-2 (F; P < 0.0001, Mann–Whitney tests) point patterns and randomly simulated data in treated groups. Experimental data DMSO control (“Ctrl,” dark gray) and after forskolin (“FSK,” dark red), randomly simulated data DMSO control (“Ctrl Null,” light gray) and after forskolin (“FSK Null,” light pink). Numerical values for this figure are detailed at https://doi.org/10.15479/AT:ISTA:18296. (TIF) [file pbio.3002879.s004.tif]

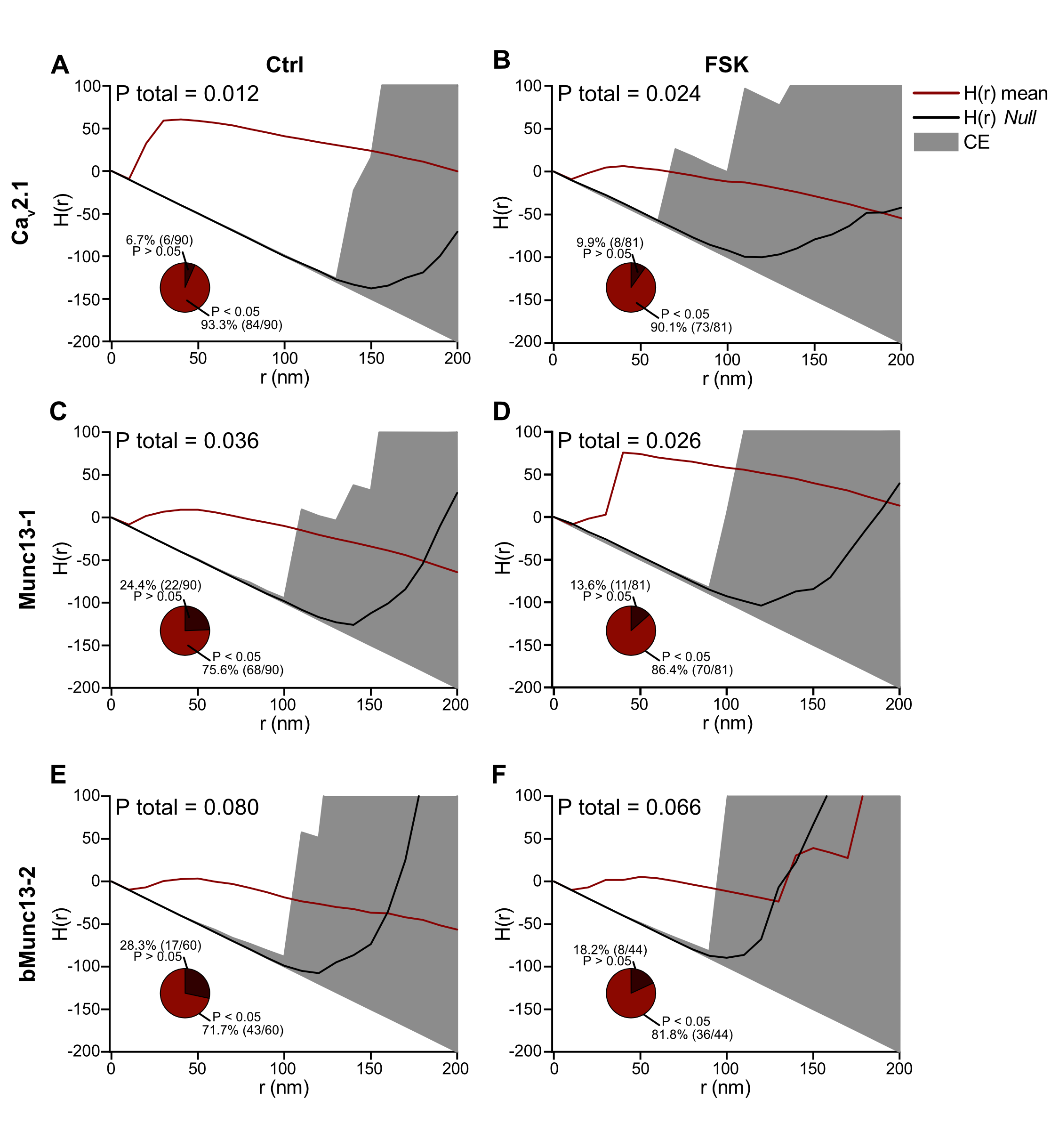

Supplement: S5 Fig — (A, B) Univariate H(r) function of CaV2.1 in DMSO control (A) and after 50 μM forskolin (B); n AZ = 90 and 81, respectively, total P-value on the figure. Red line indicates population mean, black line random distribution, and gray area—confidence envelopes (CE). Inset: pie chart of statistical significance of MAD test of single AZs. (C, D) Univariate H(r) function of Munc13-1 in DMSO control (C) and after 50 μM forskolin (D); n AZ = 90 and 81, respectively, total P-value on the figure. Red line indicates population mean, black line–random distribution, and gray area–confidence envelopes (CE). Inset: pie chart of statistical significance of MAD test of single AZs. (E, F) Univariate H(r) function of bMunc13-2 in DMSO control (E) and after 50 μM forskolin (F); n AZ = 60 and 44, respectively, total P-value on the figure. Red line indicates population mean, black line–random distribution, and gray area–confidence envelopes (CE). Inset: pie chart of statistical significance of MAD test of single AZs. Numerical values for this figure are detailed at https://doi.org/10.15479/AT:ISTA:18296. (TIF) [file pbio.3002879.s005.tif]

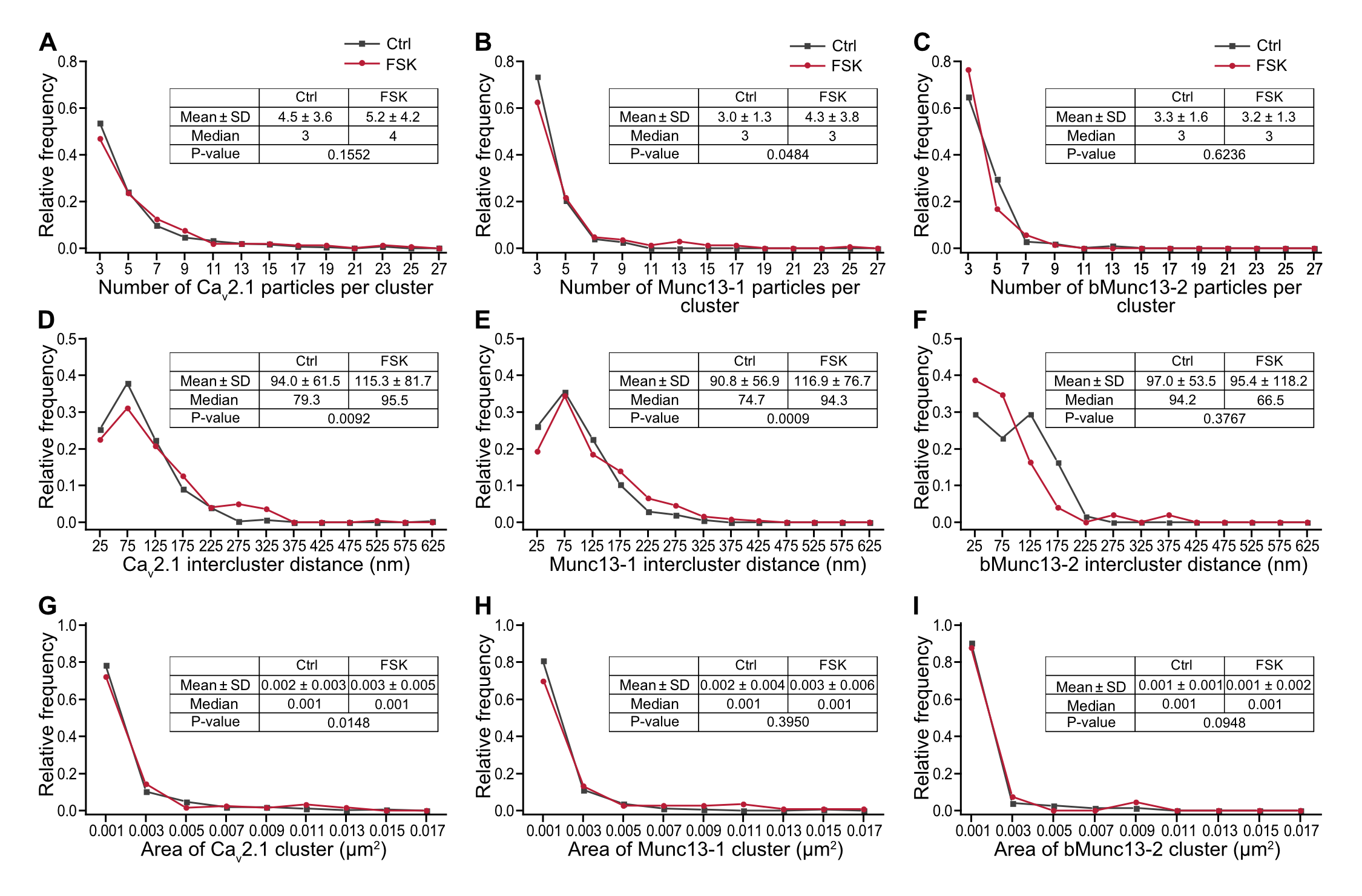

Supplement: S6 Fig — (A–C) Histograms of relative frequency distribution of number of particles per CaV2.1 (A), Munc13-1 (B), and bMunc13-2 (C) cluster in DMSO control (“Ctrl,” gray) and after 50 μM forskolin (“FSK,” red). Total P-values are indicated on the figures. (D–F) Histograms of relative frequency distribution of minimal distance between CaV2.1 (D), Munc13-1 (E), and bMunc13-2 (F) clusters, color scheme is identical to (A–C). Total P-values are indicated on the figures. (G–I) Histograms of relative frequency distribution of area of each cluster of CaV2.1 (G), Munc13-1 (H), and bMunc13-2 (I) particles, color scheme is identical to (A–C). Total P-values are indicated on the figures. (TIF) [file pbio.3002879.s006.tif]

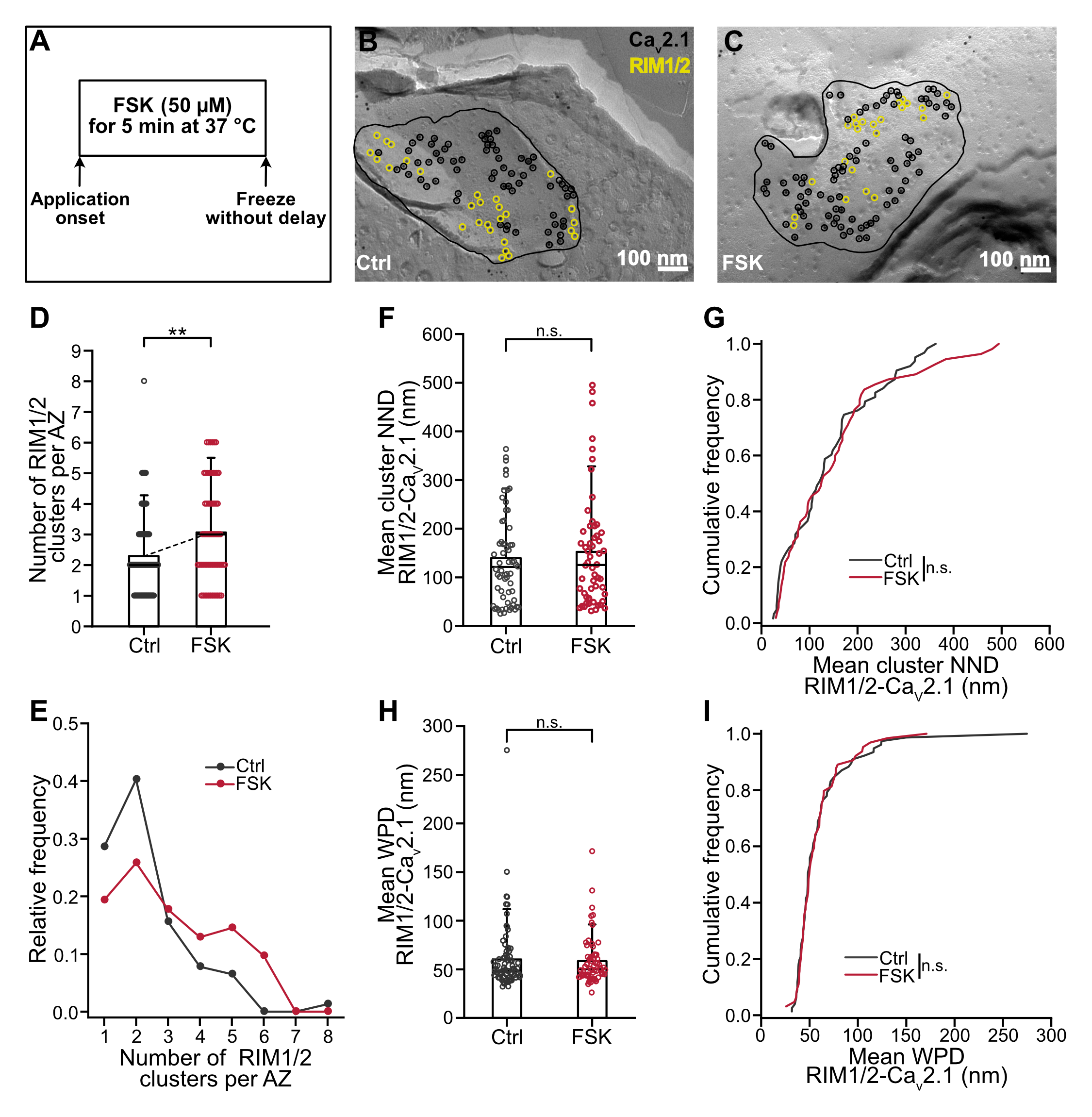

Supplement: S7 Fig — (A) Schematic representation of the time course of the experiment with 5-min forskolin (FSK) treatment. (B, C) Example TEM micrographs of freeze-fractured replica of acute slices showing putative MFB AZ (black line) co-labeled against CaV2.1 (black empty circles) and RIM1/2 (yellow empty circles) in DMSO control (B, “Ctrl”) and after 50 μM forskolin (C, “FSK”). Scale bar sizes are indicated on the figure panels. (D) Summary bar graph of the number of RIM1/2 clusters per AZ in DMSO control (“Ctrl,” gray) and after forskolin treatment (“FSK,” red). Bars and whiskers show mean + SD. Horizontal black lines indicate median values. P = 0.0042, Mann–Whitney test. (E) Relative frequency distribution of data shown in (D), color scheme is identical to (D). (F) Summary bar graph of the mean NNDs between RIM1/2 and CaV2.1 clusters in DMSO control (“Ctrl,” gray) and after forskolin treatment (“FSK,” red). Bars and whiskers show mean + SD. Horizontal black lines indicate median values. P = 0.6350, Mann–Whitney test. (G) Cumulative plots of mean NNDs between RIM1/2 and CaV2.1 clusters, color scheme is identical to (F). P = 0.6350, Mann–Whitney test. (H) Summary bar graph of the mean WPDs between RIM1/2 and CaV2.1 in DMSO control (“Ctrl,” gray) and after forskolin treatment (“FSK,” red). Bars and whiskers show mean + SD. Horizontal black lines indicate median values. P = 0.7156, Mann–Whitney test. (I) Cumulative plots of mean WPDs between experimental RIM1/2 and CaV2.1 point patterns, color scheme is identical to (H). P = 0.7156, Mann–Whitney test. Numerical values for this figure are detailed at https://doi.org/10.15479/AT:ISTA:18296. (TIF) [file pbio.3002879.s007.tif]

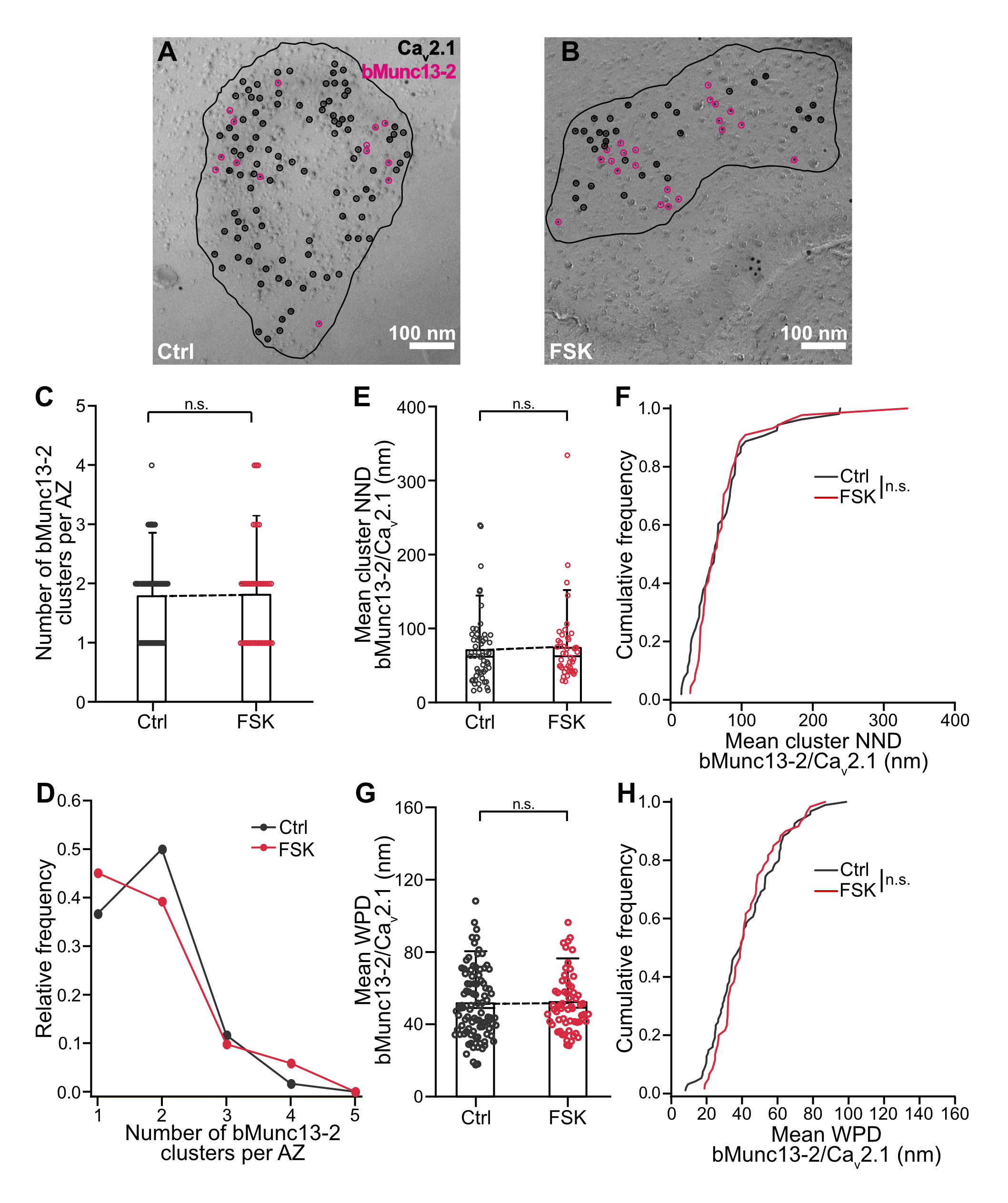

Supplement: S8 Fig — (A, B) Example TEM micrographs of freeze-fractured replica of acute slices showing putative MFB AZ (black line) co-labeled against CaV2.1 (black empty circles) and bMunc13-2 (pink empty circles) in DMSO control (A, “Ctrl”) and after 50 μM forskolin (B, “FSK”). Scale bar sizes are indicated on the figure panels. (C) Summary bar graph of the number of bMunc13-2 clusters per AZ in DMSO control (“Ctrl,” gray) and after forskolin (“FSK,” red). Bars and whiskers show mean + SD. Horizontal black lines indicate median values. P = 0.8124, Mann–Whitney test. (D) Relative frequency distribution of data displayed in (C), color scheme is identical to (C). (E) Summary bar graph of the mean NNDs between bMunc13-2 and CaV2.1 clusters in DMSO control (“Ctrl,” gray) and after FSK treatment (“FSK,” red). Bars and whiskers show mean + SD. Horizontal black lines indicate median values. P = 0.5943, Mann–Whitney test. (F) Cumulative plots of mean NNDs between experimental bMunc13-2 and CaV2.1 clusters, color scheme is identical to (E). P = 0.5943, Mann–Whitney test. (G) Summary bar graph of the mean WPDs between bMunc13-2 and CaV2.1 in DMSO control (“Ctrl,” gray) and after FSK treatment (“FSK,” red). Bars and whiskers show mean + SD. Horizontal black lines indicate median values. P = 0.6687, Mann–Whitney test. (H) Cumulative plots of mean WPDs between experimental bMunc13-2 and CaV2.1 point patterns, color scheme is identical to (G). P = 0.6687, Mann–Whitney test. Numerical values for this figure are detailed at https://doi.org/10.15479/AT:ISTA:18296. (TIF) [file pbio.3002879.s008.tif]

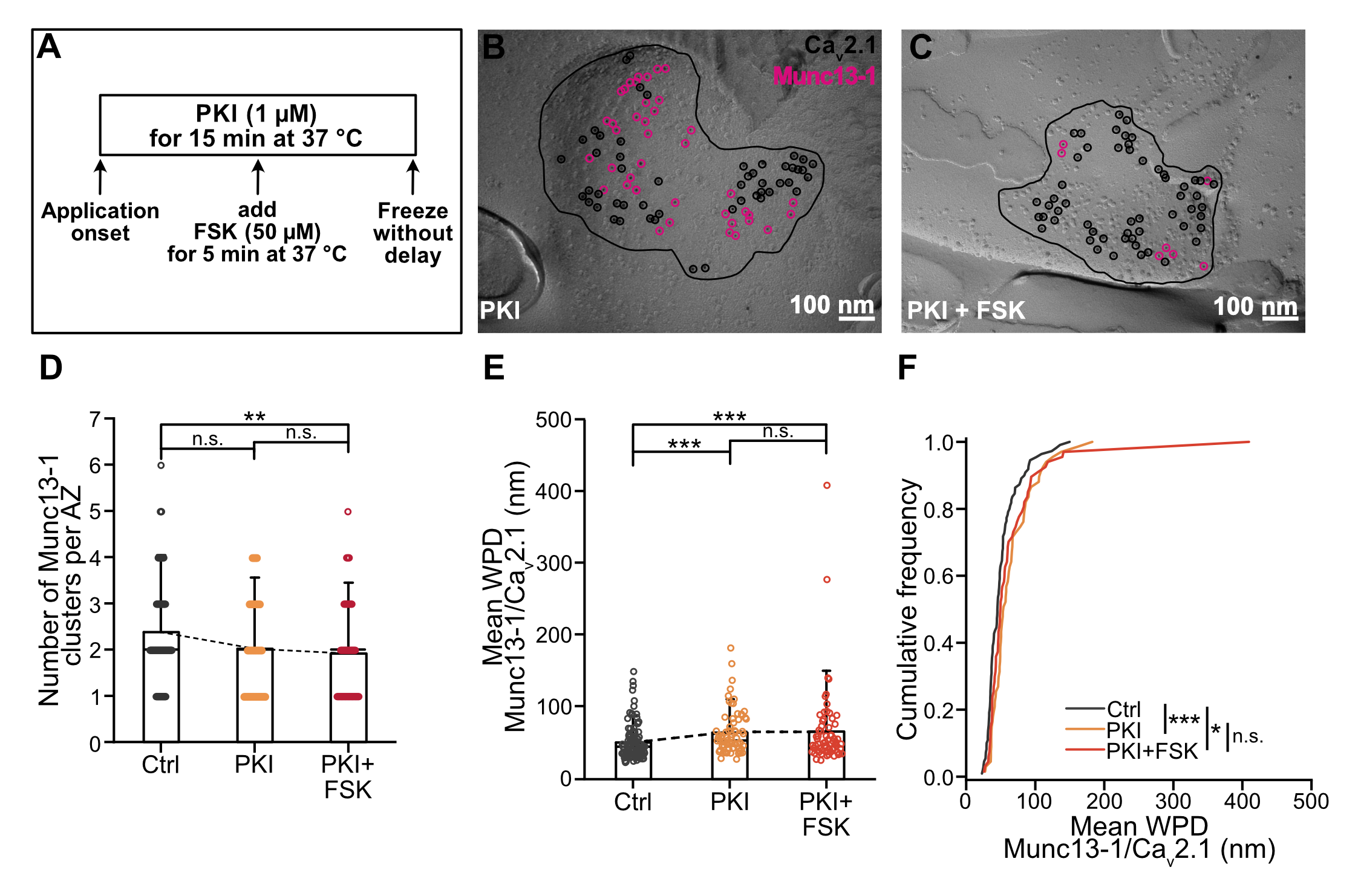

Supplement: S9 Fig — (A) Schematic representation of the time course of the experiment with 15-min PKI and 5-min forskolin (FSK) treatment. (B, C) Example TEM micrographs of freeze-fractured replica of acute slices showing putative MFB AZ (black line) co-labeled against CaV2.1 (black empty circles) and Munc13-1 (pink empty circles) in sample treated with 1 μM PKI (“PKI,” B) and after PKI with FSK treatment (“PKI+FSK,” C). Scale bar sizes are indicated on the figure panels. (D) Summary bar graph of the number of Munc13-1 clusters per AZ in DMSO control (“Ctrl,” gray) and after PKA inhibitor PKI (“PKI,” orange), and PKI with forskolin (“PKI+FSK,” red). Bars and whiskers show mean + SD. Horizontal black lines indicate median values. Control vs. PKI: P = 0.0514, control vs. PKI+forskolin: P = 0.0070, PKI vs. PKI + forskolin: P = 0.5548, Mann–Whitney test. (E) Summary bar graph of the mean WPDs between Munc13-1 and CaV2.1 in DMSO control (“Ctrl,” gray) and after PKA inhibitor PKI (“PKI,” orange), and PKI with forskolin (“PKI+FSK,” red). Bars and whiskers show mean + SD. Horizontal black lines indicate median values. Control vs. PKI + forskolin: P = 0.0009, control vs. PKI: P < 0.0001, PKI vs. PKI + forskolin: P = 0.2047, Mann–Whitney test. (F) Cumulative plots of mean WPDs between experimental Munc13-1 and CaV2.1 point patterns, color scheme is identical to (E). Control vs. PKI + forskolin: P = 0.0154, control vs. PKI: P = 0.0002, PKI vs. PKI + forskolin: P = 0.2047, Mann–Whitney test. (TIF) [file pbio.3002879.s009.tif]
