# Supplementary material for: Presynaptic cAMP-PKA-mediated potentiation induces reconfiguration of synaptic vesicle pools and channel-vesicle coupling at hippocampal mossy fiber boutons
Source: PLoS Biol. 2024 Nov 18;22(11):e3002879. doi: 10.1371/journal.pbio.3002879 (PMC11573138; doi:10.1371/journal.pbio.3002879)
Supplement: S1 Table — (PDF) [file pbio.3002879.s010.pdf]

	Number of particles per 0.1 µm² AZ	Mean	SD	Median	n AZ (N mice)	P value
**$Ca_V2.1$**	Control	36.2	12.3	35.0	130 (5)	0.3598
	Forskolin	38.8	15.3	38.8	52 (3)	
**Munc13-1**	Control	26.0	12.5	23.4	66 (3)	0.0689
	Forskolin	30.1	13.3	28.0	52 (3)	
**bMunc13-2**	Control	22.9	11.2	21.2	65 (3)	0.9543
	Forskolin	22.6	11.6	21.4	49 (3)	
	**Mean NND (nm)**					
**$Ca_V2.1$**	Control	26.6	6.2	25.9	106 (5)	< 0.0001
	Control *Null*	40.3	15.0	36.8	106	
	Forskolin	25.0	4.8	23.9	52 (3)	< 0.0001
	Forskolin *Null*	37.2	13.2	32.9	52	
**Munc13-1**	Control	37.5	32.3	28.7	99 (3)	< 0.0001
	Control *Null*	59.0	33.4	50.0	99	
	Forskolin	28.9	8.1	27.7	51 (3)	< 0.0001
	Forskolin *Null*	37.2	13.2	32.9	51	
**bMunc13-2**	Control	41.2	23.4	36.2	92 (3)	< 0.0001
	Control *Null*	69.5	39.9	59.0	92	
	Forskolin	33.7	12.0	31.3	48 (3)	< 0.0001
	Forskolin *Null*	59.2	37.1	52.2	48	
