# Supplementary material for: Presynaptic cAMP-PKA-mediated potentiation induces reconfiguration of synaptic vesicle pools and channel-vesicle coupling at hippocampal mossy fiber boutons
Source: PLoS Biol. 2024 Nov 18;22(11):e3002879. doi: 10.1371/journal.pbio.3002879 (PMC11573138; doi:10.1371/journal.pbio.3002879)
Supplement: S2 Table — (PDF) [file pbio.3002879.s011.pdf]

	Number of clusters per AZ	Mean	SD	Median	n AZ (N mice)	P value
**$Ca_V2.1$**	Control	2.7	1.1	3	139 (5)	0.1595
	Forskolin	2.8	1.4	3	81 (3)	
**Munc13-1**	Control	2.4	1.1	2	119 (5)	0.0088
	Forskolin	3.0	1.4	3	78 (3)	
**bMunc13-2**	Control	1.8	0.7	2	60 (3)	0.8124
	Forskolin	1.8	0.9	2	51 (3)	
	**Mean NND (nm)**					
**Munc13-1 + $Ca_V2.1$**	Control	97.4	83.0	70.8	83 (5)	0.0653
	Forskolin	79.1	67.2	54.2	69 (3)	
**bMunc13-2 + $Ca_V2.1$**	Control	70.7	49.2	61.8	53 (3)	0.5943
	Forskolin	73.8	52.0	62.4	44 (3)	
	**Mean WPD (nm)**					
**Munc13-1 + $Ca_V2.1$**	Control	51.2	24.2	45.3	110 (5)	0.0034
	Forskolin	40.3	12.8	38.7	85 (3)	
**bMunc13-2 + $Ca_V2.1$**	Control	51.6	19.2	49.0	94 (3)	0.6687
	Forskolin	52.2	16.1	49.1	60 (3)	
