# Supplementary material for: Presynaptic cAMP-PKA-mediated potentiation induces reconfiguration of synaptic vesicle pools and channel-vesicle coupling at hippocampal mossy fiber boutons
Source: PLoS Biol. 2024 Nov 18;22(11):e3002879. doi: 10.1371/journal.pbio.3002879 (PMC11573138; doi:10.1371/journal.pbio.3002879)
Supplement: S3 Table — (PDF) [file pbio.3002879.s012.pdf]

	Number of clusters per AZ	Mean	SD	Median	n AZ (N mice)
**Munc13-1**	Control	2.4	1.1	2	62 (3)
	H-89	2.6	1.4	2	52 (3)
	H-89 + Forskolin	2.4	1.3	2	44 (3)
	**Mean NND (nm)**				
**Munc13-1 + Ca_V2.1**	Control	97.4	83.0	70.8	83 (5)
	H-89	120.0	81.4	102.5	38 3)
	H-89 + Forskolin	136.5	110.7	92.8	31 (3)
	**Mean WPD (nm)**				
**Munc13-1 + Ca_V2.1**	Control	47.3	20.6	44.4	92 (5)
	H-89	78.4	36.9	73.0	52 (3)
	H-89 + Forskolin	51.2	26.9	44.9	44(3)
