# Supplementary material for: Presynaptic cAMP-PKA-mediated potentiation induces reconfiguration of synaptic vesicle pools and channel-vesicle coupling at hippocampal mossy fiber boutons
Source: PLoS Biol. 2024 Nov 18;22(11):e3002879. doi: 10.1371/journal.pbio.3002879 (PMC11573138; doi:10.1371/journal.pbio.3002879)
Supplement: S4 Table — (PDF) [file pbio.3002879.s013.pdf]

	Control		Chemical potentiation	
	Single AZ	Whole MFB	Single AZ	Whole MFB
**RRP SVs***	1.6	47	3.8	110
**Docked SVs♦**	13	377	21	609
**RIM1/2 clusters♠**	2.3	67	3.1	90
**Munc13 clusters♠**	4.2	122	4.8	139

* To determine the number of RRP SVs per MFB, we divided mean RRP (nA) by mean mEPSCs amplitude (pA). To estimate the number of RRP SVs per AZ, we divided the RRP SV number per MFB by 29 (average number of AZs per postnatal day 28 large MFB) [**1**].

♦ To quantify number of docked SVs per AZ, we estimated the area of single profile (0.007 μm^2) and extrapolated the number of docked SV per profile to 0.12 μm^2 (average AZ size in postnatal day 28 MFB, Rollenhagen et al., 2007). Next, we multiplied single AZ number by 29 (average number of AZs per postnatal day 28 large MFB) [1].

♠ We multiplied the single-AZ number by 29 (average number of AZs per postnatal day 28 large MFB) [**1**].

*, ♦, ♠ Number of RRP SVs was estimated in rats; numbers of docked SVs and primed SVs were determined in mice; the number of AZs and average AZ size were determined in rats [**1**]. Thus, effects of species differences on our estimates cannot be excluded.

Reference:

1. Rollenhagen A, Sätzler K, Rodríguez EP, Jonas P, Frotscher M, Lübke JHR. Structural determinants of transmission at large hippocampal mossy fiber synapses. J Neurosci. 2007;27(39):10434–10444.
